# Molecular and cytological profiling of biological aging of mouse cochlear inner and outer hair cells

**DOI:** 10.1016/j.celrep.2022.110665

**Published:** 2022-04-12

**Authors:** Huizhan Liu, Kimberlee P. Giffen, Lei Chen, Heidi J. Henderson, Talia A. Cao, Grant A. Kozeny, Kirk W. Beisel, Yi Li, David Z. He

**Affiliations:** 1 Department of Biomedical Sciences, Creighton University School of Medicine, Omaha, NE 68178, USA; 2 Department of Neuroscience and Regenerative Medicine, Medical College of Georgia, Augusta University/University of Georgia Medical Partnership, Athens, GA, USA; 3 Chongqing Academy of Animal Sciences, Chongqing 402460, China; 4 Department of Otorhinolaryngology-Head and Neck Surgery, Beijing Tongren Hospital, Capital Medical University, Beijing 100730, China; 5 Lead contact

## Abstract

Age-related hearing loss (ARHL) negatively impacts quality of life in the elderly population. The prevalent cause of ARHL is loss of mechanosensitive cochlear hair cells (HCs). The molecular and cellular mechanisms of HC degeneration remain poorly understood. Using RNA-seq transcriptomic analyses of inner and outer HCs isolated from young and aged mice, we show that HC aging is associated with changes in key molecular processes, including transcription, DNA damage, autophagy, and oxidative stress, as well as genes related to HC specialization. At the cellular level, HC aging is characterized by loss of stereocilia, shrinkage of HC soma, and reduction in outer HC mechanical properties, suggesting that functional decline in mechanotransduction and cochlear amplification precedes HC loss and contributes to ARHL. Our study reveals molecular and cytological profiles of aging HCs and identifies genes such as *Sod1, Sirt6, Jund*, and *Cbx3* as biomarkers and potential therapeutic targets for ameliorating ARHL.

## In brief

Using RNA-seq, advanced imaging, and electrophysiology, Liu et al. reveal molecular and cytological profiles of aging cochlear hair cells. Their study also suggests that a functional decline in mechanotransduction and cochlear amplification precedes hair cell loss and contributes to age-related hearing loss.

## Graphical abstract

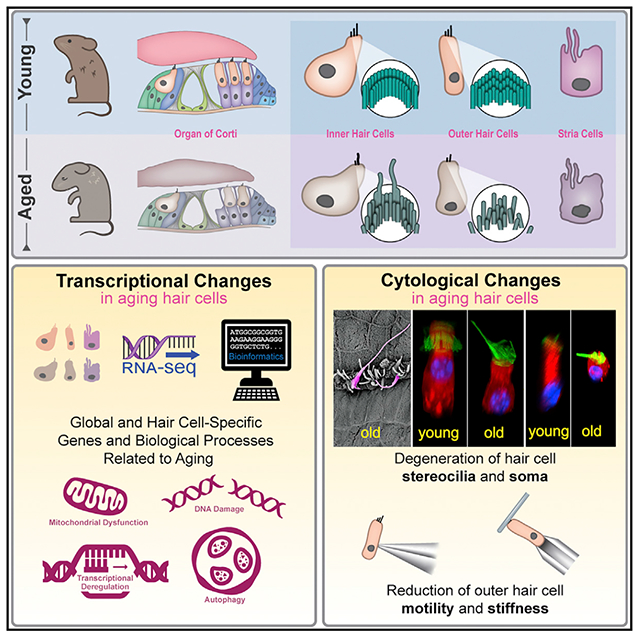

## INTRODUCTION

While many changes in the human body occur with biological aging, age-related hearing loss (ARHL) ranks among the top three chronic conditions in elderly population (Health, 1994). ARHL affects approximately one-third of adults 61 to 70 years of age and 80% of those older than 85 years (Gates et al., 1990; Mao et al., 2013). ARHL, or presbycusis, refers to the age-related changes of the peripheral and central auditory system that lead to hearing impairment and difficulty understanding spoken language. ARHL is characterized by decreased hearing sensitivity, reduced speech recognition in a noisy environment, and decreased central processing of acoustic information (Frisina, 2009). Loss of hearing in the elderly hinders the exchange of information, thus significantly impacting daily life and leading to social isolation, depression, and increased risk of dementia (Lin and Albert, 2014). Although age-related changes in the central auditory system also contribute to ARHL (Frisina, 2009), degenerative changes that lead to eventual loss of mechanosensory hair cells (HCs) in the cochlea of the inner ear is the most prevalent cause of ARHL (Ohlemiller, 2004).

The inner HCs (IHCs) and outer HCs (OHCs) are two types of sensory receptor cells in the mammalian cochlea. HCs transduce mechanical stimuli into electrical activity (mechanoe-lectrical transduction), mediated by the hair bundle in the apical surface of HCs. IHCs and OHCs have distinct morphology and function. IHCs are the true sensory receptors that transmit information to the brain, whereas OHCs serve as the effector cells that boost input to IHCs by a receptor potential-driven somatic motility (Brownell et al., 1985; Zheng et al., 2000). OHC motility, or electromechanical transduction, confers the mammalian cochlea with high sensitivity and exquisite frequency selectivity (Dallos et al., 2008; Liberman et al., 2002). Both types of HCs degenerate with age, though the molecular mechanisms are largely unknown. This fundamental lack of understanding directly impedes the development of therapeutics for intervention.

The goal of our study is to depict molecular and cellular changes associated with the biological aging of IHCs and OHCs to further elucidate the mechanism of HC aging. A fundamental understanding of molecular mechanisms driving phenotypic changes begins with a gene-level analysis. Thus, we utilized RNA-seq to analyze transcriptomes of isolated IHC, OHC, and stria vascularis cell (SVC) populations collected from cochleae of adult and aged CBA/J mice to identify aging-related transcriptional changes. Differential expression and enrichment analyses of genes related to common hallmarks of aging processes including transcription, DNA damage/repair, autophagy, and metabolic stress were examined to determine if HCs followed the same blueprint of biological aging seen in other terminally differentiated cells such as SVC, photoreceptors (Hall et al., 2017; Yi et al., 2020), and neurons (Lu et al., 2004; Ximerakis et al., 2019). We also examined cell type-specific aging signatures in IHCs and OHCs with a focus on genes related to unique HC structure and function. Furthermore, we examined cell type-specific biological processes to determine why OHCs are more vulnerable to aging than IHCs. At the cellular level we used advanced imaging and electrophysiology techniques to determine if there is an aging-related decline in mechanoelectrical and electromechanical transduction. Ultrastructural and cytological changes in HC stereocilia and soma, as well as changes in mechanical activity and property of OHCs were examined. Our study provides a comprehensive and systematic characterization of the molecular and cellular signatures of normal aging of IHCs and OHCs. Our cell type-specific transcriptomic dataset is expected to serve as a highly valuable resource for the neuroscience community to study aging processes in HCs and other sensory receptor cells in the neurosensory system.

## RESULTS

### Change in auditory function during aging

CBA/J mice were used for this study since they exhibit no hearing loss until late into their lifespan (Spongr et al., 1997; Sha et al., 2008; Ohlemiller et al., 2010), similar to human’s presbycusis. Auditory brainstem response (ABR), commonly used to determine hearing thresholds in animals, was measured at 1, 9, 18, 22, and 26 months to evaluate onset and progression of ARHL. Distortion product otoacoustic emission (DPOAE), reflecting OHC condition and function, was also measured. ABR and DPOAE thresholds of 1 months (month) and 9 months did not significantly differ (Figures S1A and S1B). At 18 months, ABR and DPOAE thresholds were significantly elevated at all frequencies. Thresholds were further elevated at all frequencies, especially at the low- and high-frequency range at 22 months. At 26 months, ABR thresholds were elevated by ~20 dB in the mid-frequency region and 35–40 dB at the low- and high-frequency regions, similar to previous studies in CBA mice (Spongr et al., 1997; Sha et al., 2008). The stria vascularis is responsible for the generation and maintaining of endocochlear potential (EP). Stria degeneration, which leads to reduction of EP, is thought to contribute to ARHL in gerbils and some mouse strains (Schulte and Schmiedt, 1992; Ohlemiller, 2006). Contrary to those findings, an increase in EP was observed in CBA mice at 26 months (Figure S1C), likely due to loss of OHCs (Liu et al., 2016, 2021). Thus, stria function is not reduced and unlikely responsible for ARHL seen in this study.

Confocal microscopy was used to evaluate HC survival along the length of the cochlea. The number of IHCs and OHCs at six cochlear locations was separately counted at 9, 22, and 26 months, with the mean count presented in Figure S1D. The percentage of surviving IHCs and OHCs at 22 months and 26 months was calculated compared to baseline HC numbers at the corresponding locations at 9 months (Figure S1E). The percentage of surviving HCs varied depending on the cochlear location (Figure S1E). There was a greater loss of HCs in the apical and basal ends of the cochleae compared to the mid-cochlear region, where most HCs remained, and a greater loss of OHCs than IHCs. HC loss was progressive, with more cell loss at 26 months than 22 months.

### Transcriptional changes in aging IHCs and OHCs

To examine changes in gene expression during aging, 1,000 IHCs and 1,000 OHCs from the upper basal (mid-cochlear) to apical turns were individually collected from cochleae of 9- and 26-month-old mice using the pipette aspiration technique (Figure S1F) (Liu et al., 2014). ABR and DPOAE thresholds at 9 months were indistinguishable from those at 1 month (Figure S1A). Importantly, influence from genes involved in HC development will be minimal. Age-matched SVCs from the inner ear were also collected for comparison because they functionally differ from HCs. Figure S1G presents an overview of the study design for cell collection and RNA-sequencing, along with representative images of isolated HCs and SVCs. Approximately 18,000 genes were detected in HCs and SVCs. After judiciously setting a cutoff for background value at 0.1 RPKM (reads per kilobase of transcript per million mapped reads), 14,542 and 12,597 genes were regarded as expressed in 9- and 26-month-old IHCs, respectively. Similarly, 14,535 and 13,856 genes were expressed in 9- and 26-month-old OHCs, respectively. For SVCs, 15,094 and 15,082 genes were respectively expressed at 9 and 26 months.

**Figure 1.**
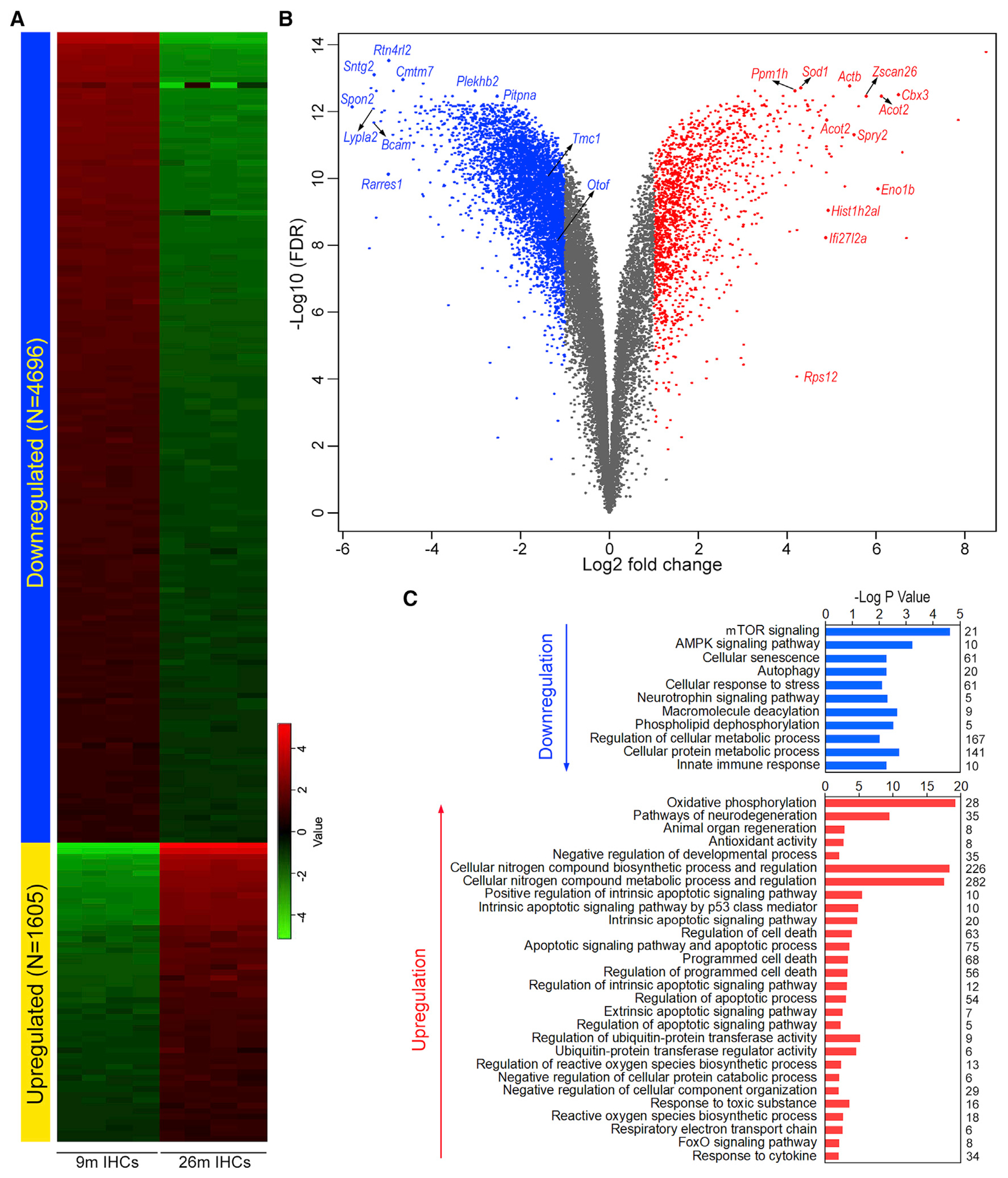
Differentially expressed gene (DEG) analysis between 9- and 26-month-old IHCs (A) Heatmap of differentially expressed genes. (B) Volcano plot of DEGs. Some of the top differentially expressed genes as well as several genes known to be related to HC function are indicated. (C) Gene set enrichment analysis of biological processes involving the top 500 up- and downregulated genes. Numeric value on the right of the panel represents the number of genes in each category.

To characterize the transcriptomic profiles underlying biological properties of aging HCs, differentially expressed genes were further analyzed. Differentially expressed genes are defined as those whose expression levels are above background with a Log2 fold change in expression ≥ |1| between the two ages (FDR p ≤ 0.10). The analysis identified 6,301 genes that were differentially expressed in 26-month-old IHCs, with 1,605 upregulated and 4,696 downregulated. In 26-month-old OHCs, 6,408 genes were differentially expressed with 3,040 upregulated and 3,368 downregulated. In both HC populations, downregulated genes outnumbered upregulated ones. Interestingly, only 3,042 genes were differentially expressed in 26-month-old SVCs with a similar number of downregulated (1,674) and upregulated (1,368) genes. Figure 1A shows the heatmap comparison of differentially expressed genes between 9- and 26-month-old IHCs, while Figure 1B presents the volcano plot of differentially expressed genes in IHCs. Gene set enrichment analysis (GSEA) including gene ontology (GO) biological processes and KEGG pathways were used to examine upregulated and downregulated IHC genes. Significantly enriched processes are shown in Figure 1C. Downregulated pathways included cellular senescence, response to stress, metabolic processes, and innate immune response. Genes involved in mTOR signaling, autophagy, and AMPK signaling pathways were also downregulated. Upregulated pathways included those related to oxidative phosphorylation, antioxidant activity, pathways of neurodegeneration, apoptotic processes and programmed cell death, reactive oxygen species biosynthesis, FoxO signaling pathways, and response to cytokine.

**Figure 2.**
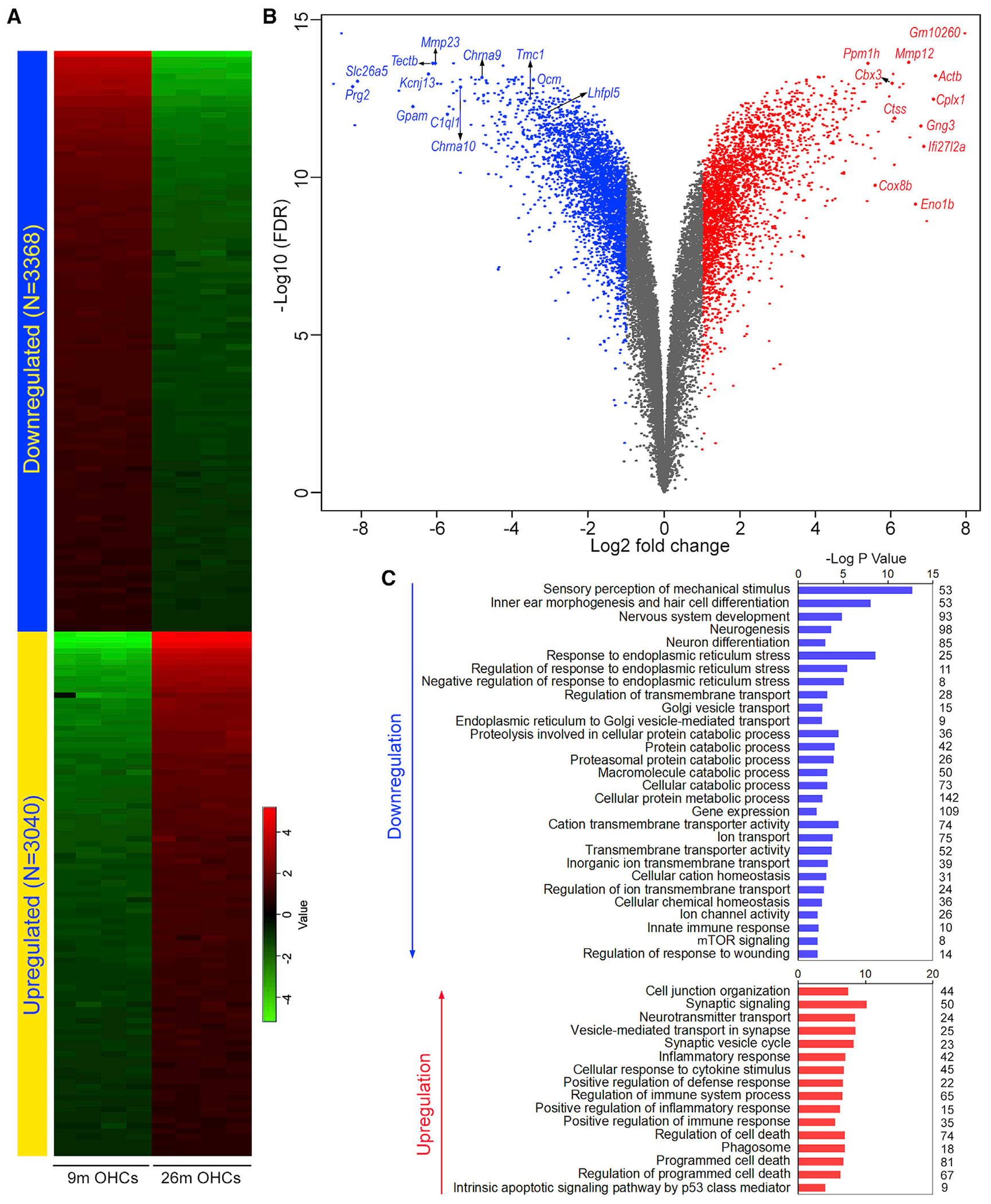
DEG analysis between 9- and 26-month-old OHCs (A) Heatmap of differentially expressed genes. (B) Volcano plot of DEGs. Some top DEGs are labeled. Some of the top differentially expressed genes as well as several genes known to be related to HC function are indicated. (C) Gene set enrichment analysis of biological processes involving the top 500 up- and downregulated genes. Numeric value on the right of the panel represents the number of genes in each category.

Similarly, Figures 2A and 2B show a heatmap and volcano plot of differentially expressed genes in OHCs. In contrast to IHCs, GSEA of OHC genes showed downregulation of processes including detection of mechanical stimulus involved in sensory perception, response to ER stress, protein catabolic process, gene expression, hemostasis, ion channel activity, and mTOR signaling (Figure 2C). Upregulated pathways included neurogenesis, junction organization, synaptic transmission, inflammatory response, phagosome, and apoptosis (Figure 2C).

**Figure 3.**
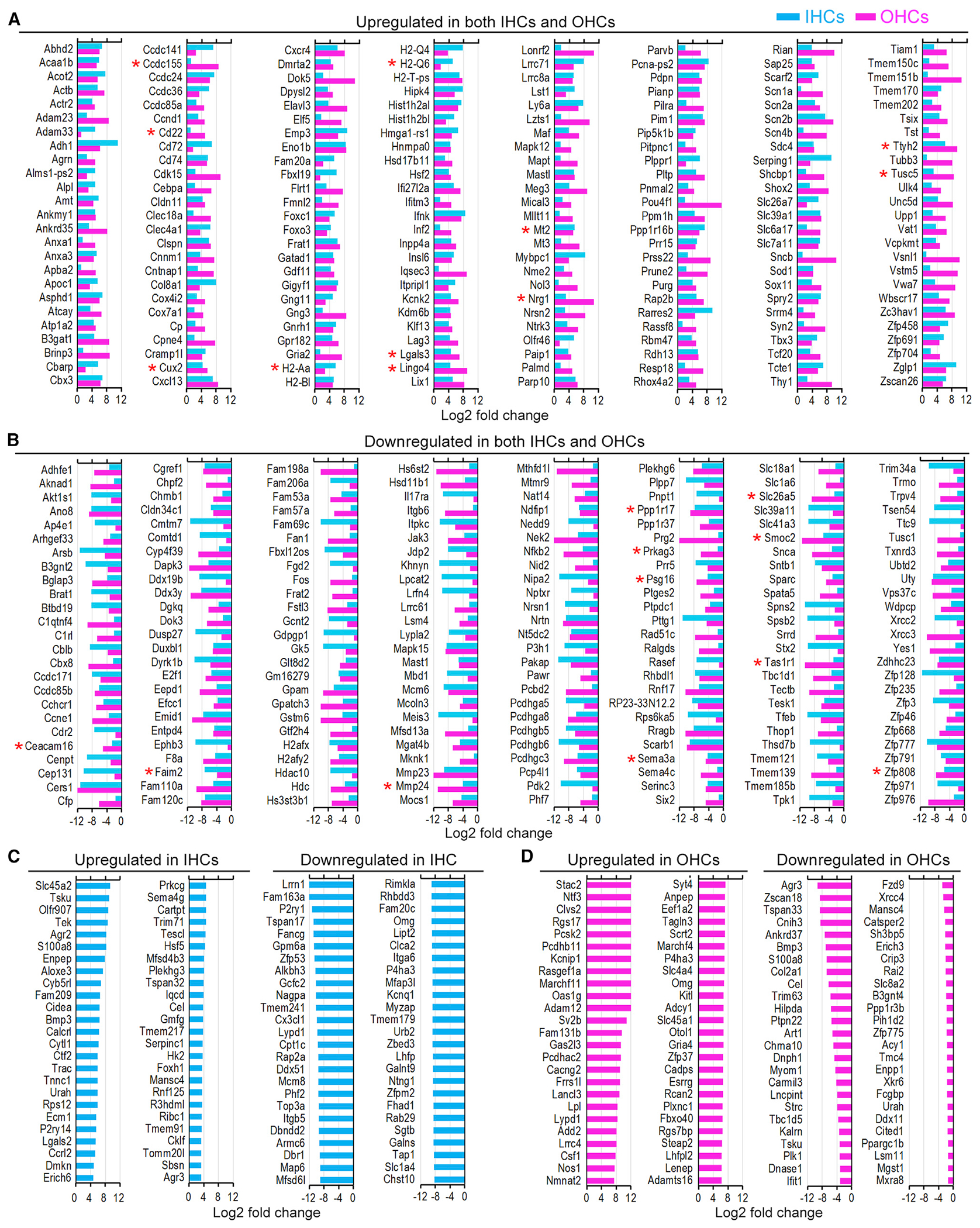
Up- and downregulated genes in aging HCs (A and B) Top 200 commonly upregulated (A) and downregulated (B) genes in both HC types at 26 months. Red asterisks indicate genes that showed the same trend of change in 26-month-old stria cells. (C) Top 50 up- and downregulated genes in 26-month-old IHCs. (D) Top 50 up- and downregulated genes in 26-month-old OHCs.

The differentially expressed genes were further analyzed to identify the top genes commonly up- or downregulated in HCs and each individual cell type during aging. Figures 3A and 3B show the top 200 up- and downregulated genes in both HC types. To determine if these genes show the same expression trend in SVCs, differential gene expression in aging SVCs was also examined. Only 22 genes (marked by red asterisks in Figures 3A and 3B) showed a similar increase or decrease in expression, suggesting most of these genes are HC specific. We also examined genes specific to only IHCs or OHCs. Figures 3C and 3D illustrate the top 50 genes whose expression was up- or downregulated only in 26-month-old IHCs or OHCs.

**Figure 4.**
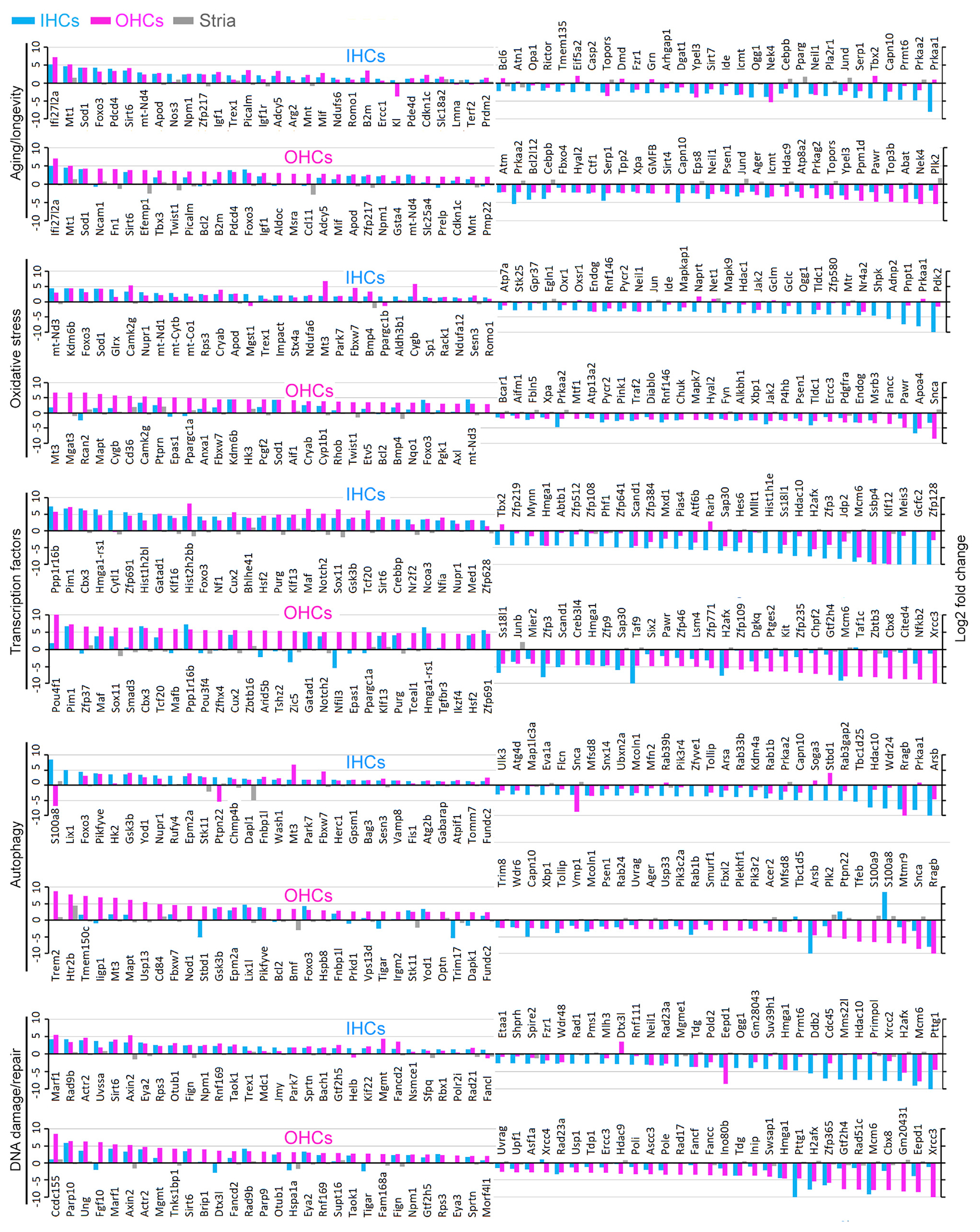
Top 30 up- and downregulated genes in IHCs and OHCs annotated in GO biological process categories of aging/longevity, oxidative stress, transcriptional regulation, autophagy, and DNA damage and repair

We next examined the expression of genes known to be associated with cellular processes of biological aging. Past studies identified genes and pathways (such as insulin/IGF-1, SIRT, and mTOR) regulating aging and longevity (van Heemst, 2010; Weichhart, 2018; Lee et al., 2019). GO annotations and GenAge database (Tacutu et al., 2018) include roughly 300 genes related to aging in mice and humans. Figure 4 presents the top 30 up- and downregulated aging-related genes in 26-month-old IHCs and OHCs. *Igf1r* (daf-2) and *Foxo3* (daf-16), two genes identified as part of the first metabolic pathway discovered to regulate the rate of aging in the worm (Kimura, 1997; Lin et al., 2001), were upregulated in both types of HCs. *Igf1* was also upregulated. The sirtuins are a class of NAD^+^-dependent deacetylases comprising seven members in mammals. *Sirt1* and *Sirt7* were downregulated in both HC types at 26 months. *Sirt3* and *Sirt5* were downregulated in IHCs, while *Sirt4* was downregulated in OHCs at 26 months. *Sirt6* is the only gene in this seven-gene family that was upregulated in both IHCs and OHCs at 26 months. *Sirt2* was highly expressed in both types of HCs with no significant change during aging. *Jund* was downregulated in both HC types. JUND is known to protect cells from p53-dependent senescence and apoptosis (Weitzman et al., 2000). Oxidative stress is another biological process associated with aging. Approximately 426 proteins participate in the oxidative phosphorylation pathway according to the KEGG pathways dataset. Figure 4 lists the top 30 up- and downregulated genes in 26-month-old IHCs and OHCs.

Transcription factors (TFs) play an essential role in the complex regulation of gene expression during aging. The change in expression of genes encoding ~1,700 TFs, including those that act as transcription co-factors or have other regulatory functions, was compared using a previous publication (Li et al., 2016). Figure 4 shows the top 30 up- and downregulated TF genes in 26-month-old IHCs and OHCs. Many TF genes such as *Pim1*, *Cbx3*, *Ppp1r16b*, *Cux*, *Sox11*, *Notch2*, *Maf*, and *Datad1* were upregulated in both types of aging HCs. Several genes such as *Klf12*, *Ssbp4*, *Mcm6*, *H2afx*, and *Taf9* shared the same trend of downregulation among the top 30 TF genes.

Autophagy and DNA damage/repair are two mechanisms involved in aging, so the expression of genes related to these processes was examined. Of the top 30 upregulated genes involved in autophagy, 11 genes (*Mt3*, *Fbxw7*, *Gsk3b*, *Epm2a*, *Pikfyve*, *Epm2a*, *Foxo3*, *Fnbp1l*, *Stk11*, *Yod1*, and *Fundc2*) were common in IHCs and OHCs (Figure 4). Four genes (*Rragb*, *Snca*, *Arsb*, and *Mfsd8*) were among the top genes commonly downregulated in both IHCs and OHCs. Interestingly, mTOR signaling, known to regulate autophagy, was downregulated in both types of HCs. Within the top 30 upregulated genes involved in DNA damage/repair (Figure 4), 12 genes (*Maf1*, *Actr2*, *Mgmt*, *Sirt6*, *Rad9b*, *Otub1*, *Taok1*, *Eya2*, *Tigar*, *Fign*, *Gtf2h5*, and *Sprtn*) were common in both HC types. For the top 30 downregulated genes, three (*H2afx*, *Pttg1*, and *Tdg*) were common in both populations of HCs.

Histone acetylation and deacetylation play a key role in the regulation of gene expression. These reactions are typically catalyzed by enzymes with histone acetyltransferase (HAT) or histone deacetylase (HDAC) activity. Downregulation of *Atf2*, *Kat7*, *Hdac2*, *Hdac5*, and *Hdac10* and upregulation of only one gene, *Hdac11*, were observed in both IHCs and OHCs. *EP300*, *Gcn5* (*Kat2a*), *Taf1*, *Elp3*, *Kat7*, *Hdac1*, *Hdac3*, and *Hdac7* were downregulated in IHCs, while *Hat1* and *Hdac9* were downregulated in OHCs at 26 months. DNA methylation, catalyzed by the DNA methyltransferases (DNMTs), is regarded as a key player in epigenetic silencing of transcription (Moore et al., 2013). DNA methylation is regulated by a family of DNMTs: DNMT1, DNMT2, DNMT3A, DNMT3B, and DNMT3L. *Dnmt1* and *Dnmt3a* expression was detected in both types of HCs; however, *Dnmt1* was downregulated in aging IHCs, while *Dnmt3a* was upregulated in aging OHCs. Both encode proteins that are important components of mammalian epigenetic gene regulation and important for maintaining methylation patterns following DNA replication.

**Figure 5.**
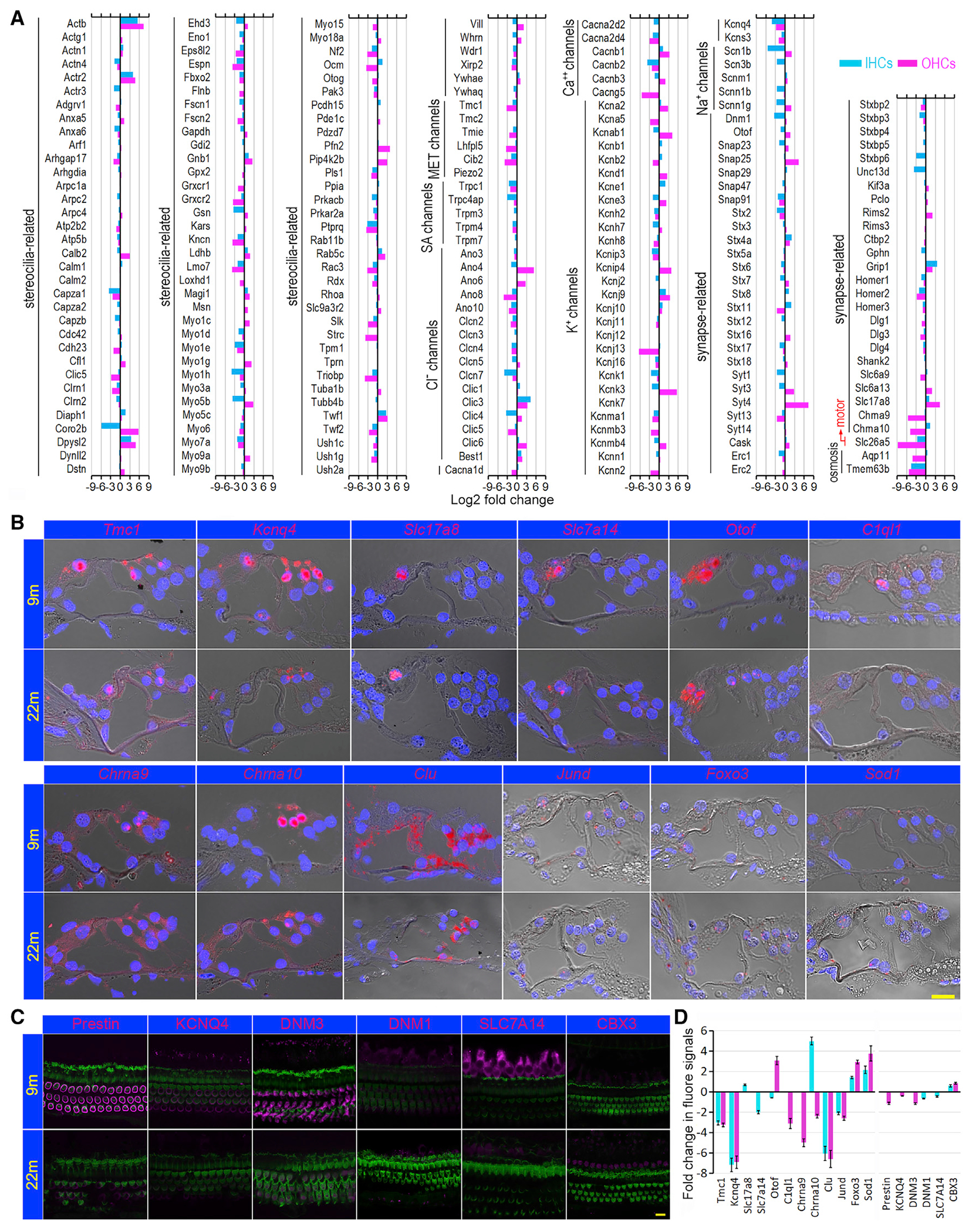
Change in gene expression during aging (A) Age-related change in expression of genes that are relate to specializations in IHCs and OHCs at 9 and 26 months. MET channels: mechanotransduction channels; SA channels: stretch activated channels. (B) Validation of change in gene expression in aging HCs using smFISH. Red signals represent the labeled mRNA transcripts (gene expression), and the nuclei were stained with DAPI (blue). (C) Validation of change in protein expression using immunostaining. Scale bars in (B) and (C) are 10 μm. (D) Mean log2 fold change in fluorescent signal (integrated density) between 9- and 22-month-old HCs (bar: ± SD) using 9 months as a baseline. Six sections from three cochleae, aged 9 or 22 months, were used for smFISH quantitative analysis. For immunostaining, whole mount preparations from three cochleae at each age were used for quantification.

HCs contain specializations in the apical and basolateral membranes for mechanotransduction, electrical potential, and synaptic transmission. Figure 5A shows the change in expression of 112 genes encoding stereocilia-associated proteins based on published mass spectrometry data (Krey et al., 2015). *Clrn1*, *Espn*, *Myo6*, *Myo7a*, *Myo15*, *Rdx*, *Ush1c*, and *Ush1g* were downregulated in both types of aging HCs. *Ocm*, *Otog*, and *Strc*, known to be preferentially expressed in OHCs (Li et al., 2018b; Liu et al., 2014), were downregulated in aging OHCs. Genes related with mechanotransduction channels and accessories, *Tmc1*, *Tmie*, *Lhfpl5*, and *Cib2* (Kawashima et al., 2011; Xiong et al., 2012; Zhao et al., 2014; Liang et al., 2021), were all downregulated in aging HCs.

Genes encoding various types of ion channels in the HC basolateral and synaptic membranes also showed changes in expression during aging (Figure 5A). Genes encoding calcium channels were all downregulated in aging HCs, except *Cacnb1* and *Cacnb3*. *Kcnq4* was downregulated in 26-month-old IHCs and OHCs, and *Kcnj13* was downregulated in aging OHCs. Interestingly, some genes (*Clic3* and *Best1*) that encode Cl^−^ channels were upregulated in both HC types, while *Ano4*, *Ano6*, and *Clic6* were upregulated in OHCs. The function of these Cl^−^ channels in HCs has not been determined.

Changes in expression of genes associated with synaptic vesicle transport and release (afferent innervation), as well as nicotinic cholinergic receptors (efferent innervation) were also examined. While many of these genes were downregulated in aging IHCs (Figure 5A), a number of genes (*Otof*, *Snap23*, *Snap29*, *Stx16*, *Syt3*, *Syt4*, *Rim2*, *Slc6a9*, and *Slc17a8*) related to afferent synapses were upregulated in aging OHCs, suggesting remodeling of the synapse. However, genes related to efferent synapses and postsynaptic density scaffolding proteins, such as *Chrna9*, *Chrna 10*, *Homer1*, *Homer2*, and *Homer3*, were downregulated in aging OHCs. Interestingly, we observed an increased expression of *Chrna10* in aging IHCs, consistent with morphological evidence that efferent synapses return to IHCs in aging cochlea (Lauer et al., 2012). *Slc26a5*, encoding motor protein prestin (Zheng et al., 2000), was downregulated by 9-fold in aging OHCs. Finally, *Tmem63b*, regulating osmosis and cell volume in HCs (Du et al., 2020), was downregulated by 5-fold. *Apq11*, differentially expressed in OHCs (Liu et al., 2014), was downregulated 4-fold in aging OHCs.

Recently, single-cell RNA-seq was used to examine changes in gene expression of OHCs and spiral ganglion neurons after noise exposure (Jongkamonwiwat et al., 2020; Milon et al., 2021). We compared these datasets with ours to determine if aging and noise-induced response share common genes and pathways. The genes that are common in both processes are presented in Figure S2. 166 genes are commonly upregulated in post-noise OHCs and aging HCs. ShinyGO analysis shows that the common upregulated biological processes include immune response (*Cd74*, *Ifitm1*, *Ifitm3*, *Lgals9*, and *Csf1*) and cellular response to stress (*Wfdc18*, *Oasic*, *Cybb*, and *Dhx58*). 109 genes are commonly downregulated in post-noise OHCs and aging HCs. The common downregulated biological processes include regulation of transmembrane transport (*Slc8a1* and *Dpp6*) and ion channels (*Cacna1b*, *Kcnb2*, and *Kcna5*). Our analysis suggests that aging and response to noise share some common processes such as inflammation and response to stress. But the changes in response to aging and noise are largely dissimilar.

Changes in gene expression between 9- and 26-month-old HCs were validated by using single molecule fluorescence *in situ* hybridization (smFISH). Twelve genes were used for comparison. *Tmc1*, *Kcnq4*, *Slc17a8*, *Slc7a14*, *Otof*, *Chrna9*, and *Chrna10* were chosen as they are vital for HC function. *Slc7a14* is expressed in IHCs, and loss of function can cause syndromic hearing loss (Giffen et al., 2022). *Clu*, *Jund*, and *Foxo3* were also included as they are involved in the regulation of aging, apoptosis, and autophagy, while *Sod1* encodes an isozyme responsible for free radical destruction. *C1ql1* (Qi et al., 2021), a gene involved in HC innervation, is also included. Example FISH images depicting mRNA-level expression are shown in Figure 5B. Immunostaining was used to examine the change in expression of six different proteins (prestin, DNM3, KCNQ4, SLC7A14, DNM1, and CBX3) (Figure 5C). The mean fold change in fluorescent signal intensity (integrated density in smFISH or intensity in immunostaining) was quantified in 9- and 22-month-old HCs (Figure 5D). As shown, the change in gene expression is consistent with the trend observed with RNA-seq.

### Cytological changes in aging HCs

**Figure 6.**
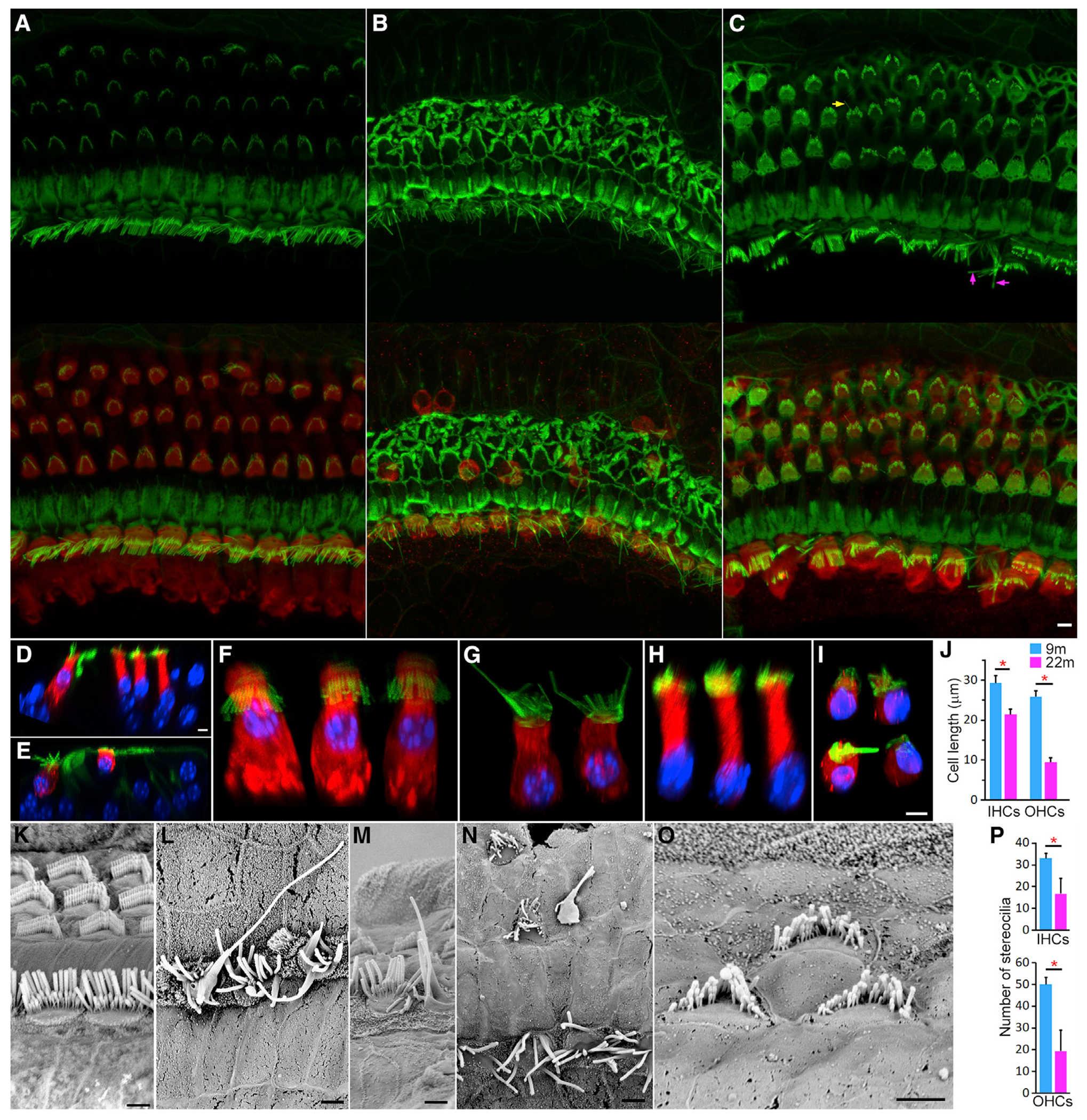
Morphological changes of HCs during aging (A) Confocal image of HCs 500 μm from the apical end of a 9-month-old cochlea. (B and C) Confocal images of HCs at cochlear locations 500 μm (B) and 1,800 μm (C) from the apical end of 22-month-old cochlea. Stereocilia were labeled with phalloidin (top panels), and HCs were stained with MYO7A (merged in bottom panels). Elongating stereocilia are marked with arrows in (C). Bar: 5 μm. (D) Cross-section of the organ of Corti from 9-month-old cochlea using confocal virtual sectioning. (E) Confocal optical section from 22-month-old cochlea. (F and G) Images of individual IHCs at 9 months (F) and 26 months (G). (H and I) Images of virtual individual OHCs at 9 months (H) and 22 months (I). Images were obtained from z stacked confocal images, following rotation of the image and manual removal of adjacent HCs and supporting cells in the background using Photoshop. (J) Mean resting cell length of IHCs and OHCs at 9 and 22 months (±SD, n = 22 for IHCs and 27 for OHCs; three mice for each age). Red asterisk marks statistical significance between the two age groups (p = 8.8E-11 and 2.0E-42 for IHCs and OHCs, respectively). (K) SEM micrograph of stereocilia bundles of the apical turn HCs at 9 months. Bar: 2 μm. (L–O) SEM micrographs of degenerating stereocilia bundles (some are highlighted in purple) at 22 months. Bars: 2 μm. (P) Comparison of the number of stereocilia of IHCs and OHCs between 9 and 22 months. Red asterisk marks statistical significance between the two age groups (p = 4.3E-07 and 2.7E-09 for IHCs and OHCs, respectively).

Confocal microscopy was used to examine HC and stereocilia bundle morphology in 22-month-old mice. We focused on HCs in the apical turn (low- and mid-frequency regions) as degeneration appearing in the apical end of the cochlea at this age is illustrative of the simultaneous degeneration in the basal end of the cochlea (Video S1), which eventually extends to mid-cochlear region. Figures 6A and 6B show representative confocal images of stereocilia and MYO7A-labeled HCs from cochlear locations roughly 500 μm from the apical end of 9- and 22-month-old cochleae. Most OHCs were lost in this region at 22 months, while the majority of IHCs were still present. Many IHCs exhibited elongation of individual stereocilia between 12 and 20 μm in length, up to five times longer than normal. In regions approximately 1.8 mm from the apex of the cochlea, elongating IHC stereocilia were only occasionally observed (Figure 6C, arrows). Sporadic loss of IHCs and OHCs was also observed.

Confocal sectioning was used to further examine HC morphology in the whole mount preparation. Representative virtual cross-section images of the organ of Corti from 9- and 22-month-old mice are presented in Figures 6D and 6E, respectively. Images of virtual individual IHCs and OHCs were obtained from z stacked confocal images, following rotation of the image and manual removal of adjacent HCs and supporting cells in the background using Photoshop. Representative images are presented in Figures 6F–6I. In comparison to 9-month-old IHCs (Figure 6F), 22-month-old IHCs were shorter in length, and the nucleus was located toward the bottom of the cell (Figure 6G). The surviving OHCs in the same region showed evidence of swelling, with cell length reduced to 9–12 μm (Figures 6E and 6I). This contrasts with the typical OHC length of 25–28 μm in this region (Figures 6D and 6H). Figure 6J presents the mean length of IHCs and OHCs measured from confocal optical section images. Both HC types exhibited a significant reduction in resting cell length, with a greater reduction in OHCs. Thus, cell shrinkage is one of the morphological features seen in aging HCs.

Scanning electron microscopy (SEM) was used to examine the morphology of the stereocilia bundles of IHCs and OHCs in the apical end of the cochlea at 9 months (Figure 6K) and 22 months (Figures 6L–6O). Elongation and fusion of stereocilia were observed in some IHCs (Figures 6L and 6M). OHC bundles showed signs of degeneration including fusion, reduced length of stereocilia on both ends of the V-shaped bundle, and reduction in the number of stereocilia (Figures 6N and 6O). The number of stereocilia per HC at 9 and 22 months was measured from SEM micrographs, and the mean (n = 28 for each cell type, from three cochleae) is presented in Figure 6P. As shown, there is a significant reduction of the total number of stereocilia in both HC types at 22 months, especially in OHCs. The change in hair bundle morphology is another notable feature in aging HCs.

**Figure 7.**
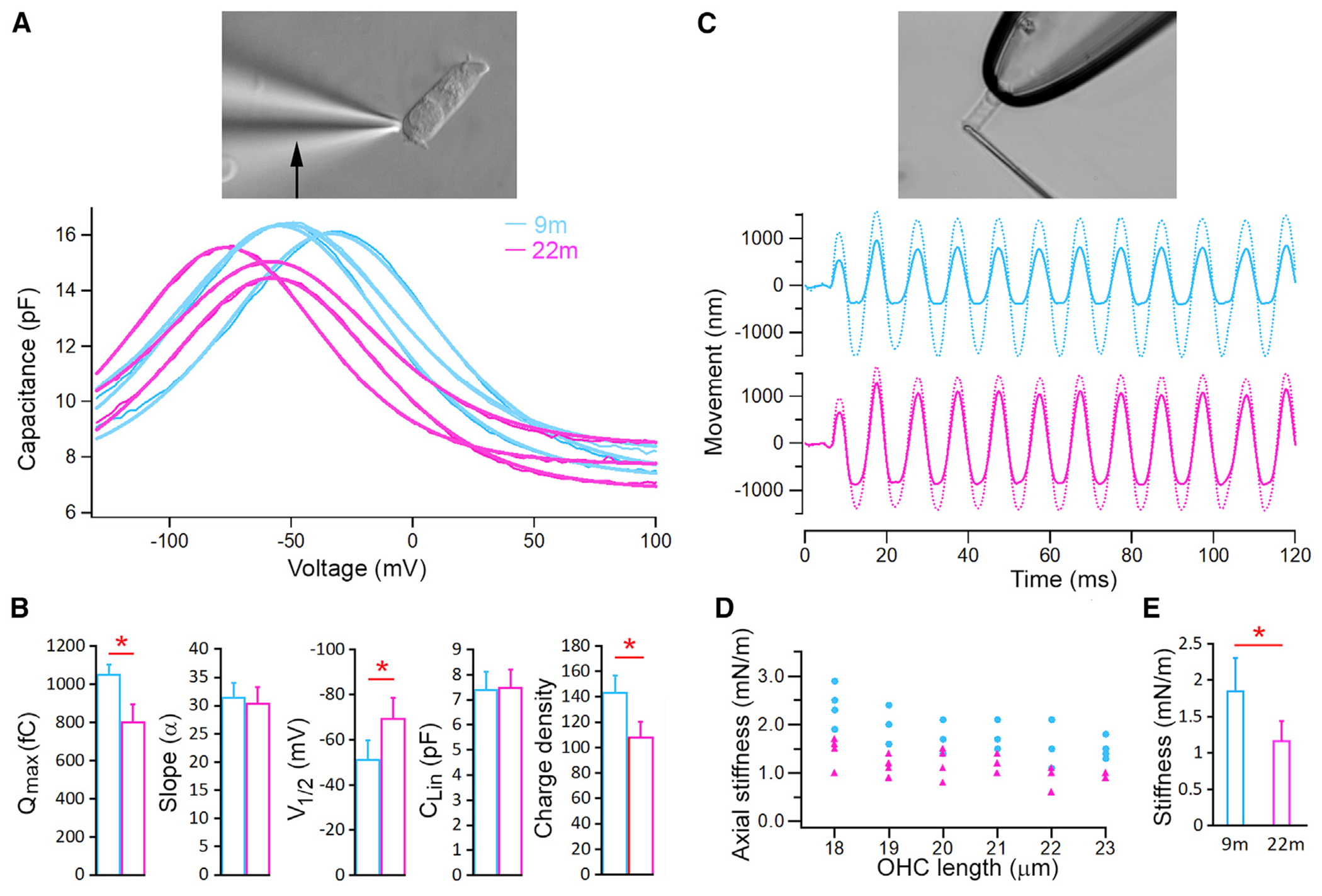
Examples of NLC response and axial stiffness measured from OHCs (A) Example NLC measurements from three 9-month-old and three 22-month-old OHCs. An image of an isolated OHC under voltage-clamp recording is shown on the top. Heavy lines in the plot are curve fitting using a two-state Boltzmann function relating nonlinear charge movement to voltage. (B) Mean values (±SD) of five parameters computed from curving fitting. Red asterisk marks statistical significance between the two age groups. p values = 1.47E-09 (Q_max_), 0.37 (slope), 5.6E-06 (V_1/2_), 0.72 (C_lin_), 4.85E-08 (density). (C) Examples of free fiber (dotted line) and loaded fiber motions (solid line) obtained from 9- and 22-month-old OHCs. Image of an isolated OHC held by a microchamber and loaded with a fiber (adopted from Dallos et al., 2008) is shown on the top panel. (D) Axial stiffness of individual OHCs and their resting length; each data point represents measurement from one OHC. (E) Mean axial stiffness (±SD) of 9- and 22-month-old OHCs. A red asterisk indicates statistical significance between the two age groups (p = 7.6E-07; n = 20 and 22 for 9- and 22-month-old OHCs, respectively).

Prestin (SLC26A5) is the motor protein of OHCs and critical for cochlear amplification (Dallos et al., 2008; Liberman et al., 2002; Zheng et al., 2000). The effects of reduced expression of *Slc26a5* on the change of OHC motility were examined. Nonlinear capacitance (NLC), an electric signature of electromotility (Santos-Sacchi, 1991), was measured from OHCs isolated from 9- and 22-month-old cochleae. OHCs with similar resting length from the two age groups were used for analysis. Figure 7A shows representative NLC responses obtained from three OHCs from 9- and 22-month-old mice, respectively. A two-state Boltzmann function (heavy lines) was used to compute four parameters (He et al., 2010). Charge density was calculated after normalizing Q_max_ with linear capacitance, which is proportional to cell size. The mean of all five parameters obtained from 9-month-old (n = 10) and 22-month-old (n = 9) OHCs is presented in Figure 7B. Statistical analysis showed that Q_max_ and charge density were significantly reduced in 22-month-old OHCs compared with those of 9-month-old OHCs, suggesting reduced prestin density and electromotility in aging OHCs.

OHC stiffness is important for both passive and active cochlear mechanics (He et al., 2003; He and Dallos, 1999). We measured axial stiffness of isolated OHCs by loading a glass fiber of known stiffness onto the apical surface of the OHC held in a microchamber (top panel in Figure 7C). Figure 7C shows some examples of free fiber motion and loaded fiber motion obtained from 9- and 22-month-old OHCs, respectively. Axial stiffness was calculated based on the stiffness of the fiber and the measurement of free fiber and loaded fiber displacement (He et al., 2003; He and Dallos, 1999). OHCs with similar cell lengths from the two age groups were chosen for comparison. Figure 7D presents axial stiffness obtained from 9-month-old (n = 20) and 22-month-old (n = 22) OHCs. The mean stiffness (±SD) from the two age groups is presented in Figure 7E. The mean axial stiffness of 22-month-old OHCs was 1.16 nN/m, significantly less than 1.84 nN/m of 9-month-old OHCs, implying that aging OHCs are more compliant.

## DISCUSSION

Aging is a highly complex trait involving many biological processes. Past studies have highlighted common hallmarks of the aging process, including genomic instability, telomere attrition, epigenetic alterations, loss of proteostasis, deregulated nutrient sensing, mitochondrial dysfunction, cellular senescence, stem cell exhaustion, and altered intercellular communication (López-Otín et al., 2013). Using cell type-specific RNA-seq analysis of HCs and SVCs from the cochleae of young and aged mice, we show that HCs exhibit both common and distinct transcriptional changes related to most of the hallmarks of aging. Key molecular processes, including transcription, DNA damage/repair, inflammation, autophagy, and metabolism, and those related to HC unique morphology and function underlie HC aging.

We observed several aging-related changes common in both types of HCs. First, the number of genes expressed in aging HCs was reduced, with downregulated genes outnumbering upregulated genes. A similar pattern is also seen in neurons (Davie et al., 2018; Ximerakis et al., 2019). Interestingly, the number of downregulated and upregulated genes was similar in SVCs, suggesting that HCs and SVCs age differently. Reduction in the total number of expressed genes and prevalence of downregulated genes suggest aging-related changes in transcriptional activity, likely associated with epigenetic silencing. We observed differential expression of many genes in aging HCs involved in DNA damage and repair, as well as histone deacetylases (HDACs), histone acetyltransferases (HATs), and DNA methylation. DNA damage, which accelerates genomic instability (Vijg and Suh, 2013), is among the most widely studied transcriptional signatures of aging (Lu et al., 2004; The Tabula Muris Consortium et al., 2020; Zhang et al., 2021). Most of the HDAC-related genes were downregulated, except *Hdac11*, which was upregulated. Interestingly, 13 genes evolved in histone acetylation and deacetylation, as well as DNA methylation, were downregulated in IHCs. In contrast, seven genes were downregulated in OHCs. *H2ax* (*H2afx*) was downregulated by more than 5-fold in both HC types. *Mcm6*, encoding one of the highly conserved mini-chromosome maintenance proteins that are essential for the initiation of eukaryotic genome replication, was also downregulated in both types of HCs. The change in expression of many of these genes involved in genomic regulation and stability is highly concordant in various aging tissues (The Tabula Muris Consortium et al., 2020; Zhang et al., 2021).

Second, we observed change in expression of genes classified as TFs (Figure 4). Several upregulated TF genes in both types of HCs deserve some discussion. *Cbx3* encodes a protein that is a component of heterochromatin and is regarded as a hallmark of repressed/silenced regions of the genome (Smallwood et al., 2012). It is also involved in DNA repair, gene regulation, and telomere function (Kwon and Workman, 2008). *Cbx3* appears to be a unique molecular hallmark with increased nuclear expression in aging IHCs and OHCs (Figure 5C). *Foxo3* belongs to the forkhead family of TFs and is a master regulator of target genes that can promote longevity functions including autophagy and apoptosis (Morris et al., 2015). In the cochlea, *Foxo3* maintains auditory synapse function and is upregulated in a function-dependent manner (Gilels et al., 2013). However, the role of FOXO3 in aging HCs has not been examined. Interestingly, *Notch2* and *Sox11*, which are known to be downregulated during HC maturation, were upregulated in aging HCs, perhaps for maintaining HC specialization (Li et al., 2018a) or in response to inflammatory stimuli and alteration in hemostasis (Balistreri et al., 2016). Other TF genes that were upregulated in both HC types include *Cux2*, *Hmga1-rs1*, *Klf13*, *Maf*, and *Pim1*; the function of these genes in HCs has not been studied. Differential expression of these TF genes, encoding transcription factors and other regulatory proteins, is likely associated with changes in gene expression in aging HCs.

Third, we observed differential expression of genes related to pro-survival, pro-apoptotic, and pro-inflammatory pathways in both types of aging HCs. Pro-survival pathways, such as autophagy and mTOR signaling, were downregulated in aging HCs. *Rragb* was downregulated in both HC types along with other known autophagy genes *Rab1b* and *Rab33b* (Ao et al., 2014). *Rragb* encodes a guanine nucleotide-binding protein that plays a crucial role in the cellular response to amino acid availability through regulation of the mTORC1 signaling cascade in autophagy (Sancak et al., 2008). This decreased gene expression hints at dysfunction in autophagy as a mechanism in aging HCs. Genes involved in pro-inflammatory pathways such as *Trem2* and *Ifi27l2a* were upregulated in aging HCs. Upregulation of *Trem2* and *Ifi27l2a* is reported in microglia during aging and has been implicated in inflammatory neurodegenerative diseases associated with aging (Hammond et al., 2019). Our study suggests that aging-related degeneration of HCs involves a shift in the balance in gene expression from pro-survival, anti-apoptotic pathways toward pro-apoptotic, pro-inflammatory pathways.

In addition to those universal transcriptional changes that are also seen in other cell types during aging, HCs exhibited distinct aging signatures. The molecular signature includes downregulation of genes such as *Lhfpl5*, *Tmc1*, *Tmie*, *Cib2*, *Kcnq4*, *Myo15*, *Cdh23*, and *Slc26a5* (Figure 5). These genes are critical for HC unique functions and associated with a deafness phenotype (Kubisch et al., 1999; Liberman et al., 2002; Kawashima et al., 2011; Xiong et al., 2012; Zhao et al., 2014; Liang et al., 2021). We speculate that their reduced expression may trigger HC death after the decline reaches a deleterious tipping point because loss of function of any of these genes is detrimental to HC survival (Kubisch et al., 1999; Dallos et al., 2008; Xiong et al., 2012; Zhao et al., 2014; Liang et al., 2021). One of the cytological hallmarks of aging HCs is the stereocilia degeneration. The degeneration is manifested by elongation, fusion, and loss of stereocilia. Degeneration may be due to dysregulation of actin biosynthesis and transportation and/or genes that control bundle length. Maintenance of mature stereocilia length requires a balance of actin capping and severing (Narayanan et al., 2015). Several proteins with barbed-end actin capping activity have been identified in hair bundles (Barr-Gillespie, 2015). We found genes that relate to capping such as *Eps8*, *Eps8l2*, *Gsn*, *Twf2*, *Capza1*, *Capza2*, and *Capzb* were all downregulated. Myosin molecules also play critical roles in promoting and regulating stereocilia length (Belyantseva et al., 2005; Stepanyan and Frolenkov, 2009; Manor et al., 2011). Most myosin genes were downregulated in aging HCs.

IHCs and OHCs exhibit differential vulnerability to aging (Spongr et al., 1997; Sha et al., 2008), though the molecular mechanism is unclear. We observed a distinct difference in gene expression profiles between IHCs and OHCs during aging. Several genes involved in regulation of transcription, DNA damage/repair, autophagy, and oxidative stress were uniquely expressed in each HC type. For example, *Ccdc155*, *Xrcc3*, *Trem2*, *Usp13*, *Trem2*, *Ligp1*, *Nod1*, *Cd44*, *Cd84*, *Ptpn22*, and *Tfeb* were only altered in OHCs. Interestingly, genes important for development and differentiation such as *Pou4f1*, *Arid5b*, and *Pou3f4* were upregulated, while *Ikzf2*, *Zbtb3*, and *Taf1c* were downregulated in OHCs. Pathways for inflammatory response and positive regulation of inflammatory response and immune system process were upregulated in OHCs. Comparison of transcriptomes of adult (1-month-old) HCs show that IHCs have higher expression of anti-apoptotic genes such as *Bcl2* and *Bcl6* than OHCs (Liu et al., 2014; Li et al., 2018b). In addition, GSEA shows downregulation of processes related to mechanotransduction, macromolecule catabolic process, ion transport and ion channel activity, and cellular chemical homeostasis in aging OHCs. These differences may underpin differential vulnerability of OHCs and IHCs.

The process of aging and senescence often leads to a general decline in function of the different organ systems. For example, aging leads to decline in synaptic transmission of neurons, which drives cognitive decline (Lu et al., 2004). It is often thought that loss of HCs, afferent synapses, and spiral ganglion neurons is the main cause of ARHL (Ohlemiller, 2004; Sergeyenko et al., 2013). However, we observed degeneration of stereocilia bundle and shrinkage of soma in aging HCs, as well as reduction in axial stiffness and prestin density in aging OHCs. Degeneration of HC stereocilia bundles during aging has been reported in humans (Wu et al., 2020) and mice (Bullen et al., 2020; Wu et al., 2020). Degeneration of stereocilia bundles affects mechanotransduction, which reduces the receptor potential that drives the release of neurotransmitter and OHC motility. Reduced OHC stiffness is likely to alter cochlear mechanics. Shrinkage of HC soma alters passive cochlear mechanics, while decreased prestin density reduces OHC motility. Cytological deterioration is likely augmented by downregulation of genes (*Lhfpl5*, *Tmc1*, *Tmie*, *Cib2*, *Tmem63b*, and *Slc26a5*) related to mechanotransduction and OHC mechanical property. Collectively, our study shows that deterioration in ultrastructure and function of HCs precedes HC loss. Such decline in mechanoelectrical and electromechanical transduction is certain to lead to ARHL.

This study is a comprehensive and in-depth characterization of the molecular and cellular signatures of biological aging in cochlear HCs. Our study compellingly suggests that HC aging is driven by key molecular processes, including transcriptional regulation, DNA damage/repair, autophagy, and inflammatory response, as well as those related to HC unique morphology and function. At the cellular level, HC aging is characterized by loss of stereocilia, shrinkage of HC soma, and reduction in OHC mechanical properties. The decline in mechanotransduction and OHC electromotility accelerates HC degeneration and contributes to ARHL. From a translational perspective, aging-related genes identified such as *Foxo3*, *Jund*, *Sirt6*, *H2afx*, *Hdac10*, *Mcm6*, and *Cbx3* are also potential biomarkers and therapeutic targets for ameliorating ARHL.

### Limitations of the study

There are several limitations of our study. First, this study examined changes in gene expression in HCs during aging. Changes in mRNA levels do not necessarily equate to changes in protein expression. Although we confirmed the change in expression of six genes at the protein level with immunostaining, it is unclear if the transcriptomic changes discussed here are similarly altered in the IHC and OHC proteomes. Second, although several of the identified genes and pathways in our analysis of aging HCs have previously been shown to be associated with aging, it is not clear if they are responsible for aging processes or secondary to other mechanisms. Future studies, using gene manipulating techniques, are needed to determine their roles in HC aging. Third, HCs, especially OHCs, in the cochlea are tonotopically organized with some differences in biological properties and gene expression. We note that HCs in our study were isolated from the mid-cochlear region and the apical turn, mostly due to difficulties of isolating and maintaining aging HCs from the basal turn of the cochlea. While we did not examine changes in gene expression of HCs populations isolated from distinct cochlear locations, future applications of this approach may elucidate the molecular mechanisms underlying the increased vulnerability of HCs in the very basal and apical ends of the cochlea to aging.

## STAR★METHODS

### RESOURCE AVAILABILITY

#### Lead contact

Further information and requests for resources and reagents should be directed to and will be fulfilled by the lead contact, David Z. He (hed@creighton.edu).

#### Materials availability

This study did not generate new unique reagents.

#### Data and code availability

- The raw RNA-seq data (Fastq files), together with the processed dataset (in Excel format), are publicly available from the National Center for Biotechnology Information-Gene Expression Omnibus (GEO) (GEO accession number: GSE153882 (HCs) and GSE154833 (SVCs)).
- The analysis conducted for this study did not use any original code.
- Any additional information required to re-analyze the data reported in this paper is available from the lead contact upon request.

### EXPERIMENTAL MODEL AND SUBJECT DETAILS

Male and female CBA/J mice, purchased from the Jackson Laboratory, were reared in our animal care facility to various ages (1,9, 18, 22 and 26 months). The acoustic environment of the animal room was examined by a sound level meter (CEL-500, Casella Cel, UK). The sound levels were 68 dB SPL over 98% of the time, and 75 dB SPL 1–2% of the time. Care and use of the animals in this study were approved by the Institutional Animal Care and Use Committee of Creighton University.

### METHOD DETAILS

#### Auditory function measurements

ABR-based hearing thresholds were measured from male and female CBA mice aged between 1 and 26 months. All records were obtained in a sound-proof chamber. The mouse was anesthetized with a mixed dose of ketamine/xylazine. The body temperature was maintained at 37°C with a heating pad. ABRs were recorded in response to tone bursts from 4 to 50 kHz using standard procedures described previously (Liu et al., 2021). ABR signals were collected with subcutaneous platinum needle electrodes placed at the vertex, mastoid prominence, and leg. Response signals were amplified (100,000x), filtered, and acquired by TDT RZ6 (Tucker-Davis Technologies, Alachua, FL). Each averaged response was based on 200 stimulus repetitions. Threshold was defined visually as the lowest sound pressure level (in decibel) at which any wave (wave I to wave IV) was detected and reproducible above the noise level.

The DPOAE at the frequency of 2f_1_ -f_2_ was recorded in response to f_1_ and f_2_, with f_2_/f_1_ = 1.2 and the f_2_ level 10 dB lower than the f_1_ level. The sound pressure obtained from the microphone in the ear-canal was amplified, and Fast-Fourier transforms were computed from averaged waveforms of ear-canal sound pressure. The DPOAE threshold was defined as the f_1_ sound pressure level (measured in decibels) required to produce a response above the noise level at the frequency of 2f_1_-f_2_.

For recording the EP, a small hole in the basal turn was made using a fine drill. A glass capillary pipette electrode (10 MU) was mounted on a hydraulic micromanipulator and advanced until a stable positive potential was observed. The signals were filtered and amplified under current-clamp mode using an Axopatch 200B amplifier (Molecular Devices, San Jose, CA) and acquired by software pClamp 9.2. The sampling frequency was 10 kHz.

#### Cell isolation, cDNA preparation, and RNA-sequencing

Male and female CBA mice aged 9 and 26 months were used for gene expression analysis. The basilar membrane, together with the organ of Corti, were dissected from the cochleae and transferred to an enzymatic digestion medium in a small Petri dish. The enzymatic digestion medium contained 1 mL L-15 and 1 mg Collagenase IV (Sigma). After 5 min incubation at room temperature, the tissue was transferred to a small plastic chamber (0.8 mL in volume) containing enzyme-free culture medium (Leibovitz’s L-15, 7.35 pH, 300 mOsm). HCs were separated by enzymatic digestion (Collagenase IV from Sigma) and mechanical trituration by a 200 mL Eppendorf pipette tip. The chamber containing the HCs was then mounted onto the stage of an Olympus IX71 inverted microscope (Olympus, Japan) equipped with a video camera. The chamber with inlet and outlet was perfused with fresh L-15 medium to wash out debris for 2 min. To collect solitary HCs, two glass pipettes, each with a diameter of ~30 μm, were used to pick up IHCs or OHCs. IHCs and OHCs were identified based on their distinct morphology (Figure S1G). The pickup pipette was fabricated from 1.5 mm thin-wall glass tubing pulled by a two-stage electrode puller. The pipettes are mounted in two separate electrode holders mounted on two Leitz 3-D micromanipulators (Leitz, Germany). By moving the pickup pipette and the stage of the microscope, cells were positioned near the tip of the pipette. The suction port of the pipette holder was connected to a micrometer-driven syringe to provide positive or negative pressure in order to draw in or expel the cells (Video S2). After ~20–30 cells had been drawn into the pipette, the pipette was lifted out from the bath and quickly transferred to a microcentrifuge tube containing 50 μL RNA*later* (Thermo Fisher Scientific, Waltham, MA) to stabilize and protect RNA in HCs. Cells were expelled from the pipette by applying positive pressure. We repeated the steps for picking up and expelling HCs until approximately 50–80 IHCs and 100–120 OHCs were collected from each mouse. The whole process from dissection to completion of cell collection took less than an hour for each mouse (most HCs were submerged in RNA*later* in 30–40 min after the mouse was euthanized). This ensured minimal degradation of RNA of our samples. To collect approximately 1,000 OHCs and 1,000 IHCs from the 9m- and 26m-old mice, 10 and 18 mice were used for each biological repeat for each age group. Approximately 70 mice were used for three biological replicates for the two age groups. Cells from stria were also isolated using the same enzymatic digestion and trituration, and collected using the same technique. SVCs were composed of three cell types including basal, intermediate (melanocyte) and marginal cells.

SMART-Seq v4 Ultra Low Input RNA Kit (TakaRa Bio USA Inc, San Jose, CA) was used for generating high-quality cDNA directly from 1 to 1,000 intact cells. Genome-wide transcriptome libraries were prepared from two biological replicates with two technical repeats for each cell type and age using the Nextera Library preparation kit (Illumina, Inc., San Diego, CA). Prior to sequencing, an Agilent 2100 Bioanalyzer and a Qubit fluorometer (Invitrogen, Thermo Fisher Scientific) were used to assess library size and concentration. Transcriptome libraries were sequenced using the HiSeq 2500 Sequencing System (Illumina, San Diego, CA). Three samples per lane were sequenced, generating approximately 100 million, 100 bp paired-end reads per sample. The files from the multiplexed RNA-seq samples were demultiplexed, and fastq files were obtained. All our sequencing runs exceeded a Phred quality score of 30, which reflects a 99.9% accuracy of the correct base at a given nucleotide in the sequence and suggests that the RNA-sequencing performed was of high quality and unambiguous (Ewing et al., 1998).

To individually map the reads (paired-end reads) to the mouse genome (mm10, build name GRCm38), we used CLC Genomics Workbench software (CLC bio, Waltham, MA). Reads were mapped to exonic, intronic, and intergenic sections of the genome. Gene expression values were quantified and normalized as RPKM. The biological replicate and mean RPKM gene expression values from 9-month and 26-month-old IHCs and OHCs are included in Table S1.

#### Bioinformatic analyses

iDEP 0.93 (http://bioinformatics.sdstate.edu/idep/) was used to analyze differential expression of the genes. The expressed genes were examined for enrichment using ClueGO v. 2.5.7 (Bindea et al., 2009). Enriched biological processes and molecular functions, classified according to gene ontology (GO) terms, as well as KEGG pathways were examined (FDR p value cutoff <0.10). For reference and verification, additional resources such as the Ensembl database, gEAR (www.umgear.org) and AmiGO (http://amigo.geneontology.org/amigo) were also used. We also compared the transcriptomes of aging HCs to those of cochlear cells post-noise exposure. RNA-seq data from a recent publication (Milon et al., 2021) was analyzed in gEAR and DEG lists were downloaded.

#### Immunocytochemistry and HC count

The mice were anesthetized with a ketamine/xylazine mixture administered via intraperitoneal injection. Once the animal reached a surgical plane of anesthesia, the animal was transcardially *perfused* with 4% PFA in PBS. After perfusion, the cochlea was dissected. A small hole was made in the apex of the cochlea and round window was also ruptured by a small forcep. The cochlea was placed in 4% PFA in PBS solution and stored in 4°C refrigerator over night. After the basilar membrane together with the organ of Corti were dissected out, the tissue was then incubated for 24 h at 4°C with primary antibodies [(DNM1 (Novus NBP2-48950, 1:200), DNM3 (gift from Dr. Pietro De Camilli at Yale University, 1:200), KCNQ4 (Sigma-Aldrich, HPA018305, 1:200), CBX3 (Novus, NBP1-83228, 1:200), SLC7A14 (Sigma-Aldrich, HPA045929, 1:400) and SLC26A5 (gift from Dr. Jing Zheng at Northwestern University, 1:400 dilution)], then washed three times with 1X PBS. The secondary antibody (1:500) (Alexa Fluor 488; Invitrogen) (Life Technologies, Lot# 1579044) was added and incubated overnight at 4°C. Tissues were washed with PBS and mounted on glass microscopy slides with antifade solution (5 mL PBS, 5 mL glycerol, 0.1 g n-propylgallate). Images were captured using Zeiss 710 Confocal Microscope. For HC count, HCs were stained with anti-MYO7A antibody (Proteus, 25–6790, 1:300 dilution). Images from six areas (each with 400 μm in length) at measured locations along the length of the cochlea were captured, and IHCs and OHCs were counted separately from confocal images offline, as described previously (Liu et al., 2021). Each location was measured as the relative distance from the apical end of the basilar membrane. Overall length of the organ of Corti from the apex to base was ~5.6 mm. Three cochleae from three animals were used for each location for cell count. To obtain virtual sections of the cochlea (Figures 6D and 6E), Imaris was used to rotate and section the image. Each section was ~10 μm. For virtual images of individual cells shown in Figures 6F–6I, z-stacked confocal images were rotated to an angle so that both stereocilia and soma were clearly visualized. Adjacent HCs and supporting cells in the background were then manually erased using Photoshop.

#### RNAscope

Single molecule fluorescence *in situ* hybridization (smFISH) was used to examine the expression of 10 genes in 10 mm-thin cochlear sections. The cochlea was prepared as described above for immunocytochemistry. Samples were prepared in PFA-fixed paraffin embedded tissue. Methods for the RNAscope™ 2.5 HD Assay from Advanced Cell Diagnostics were followed. Probes for 12 genes were purchased from ACD (*Tmc1* (Cat No: 520911-C2), *Chrna9* (818511), *Kcnq4* (707481), *Chrna10* (818521), *Slc17a8* (549991), *Slc7a14* (544781), *Otof* (485678), *Clu* (427891), *Jund* (1031951-C1), *Foxo3* (485321), *C1ql1* (465081-C2), and *Sod1* (428581)). To compare the expression of gene of interest between 9 and 26 m, intensity (gray value) and area of the fluorescent signals were measured to obtain integrated density for individual IHCs and OHCs in each cochlear section. Fluorescent signals from three sections were averaged for each cell type at each age.

#### SEM

The cochleae from young and aging mice were fixed for 24 h with 2.5% glutaraldehyde in 0.1 M sodium cacodylate buffer (pH 7.4) containing 2 mM CaCl_2_, washed in buffer. After the cochlear wall was removed, the cochleae were then post-fixed for 1 h with 1% OsO_4_ in 0.1 M sodium cacodylate buffer and washed. The cochleae were dehydrated via an ethanol series, critical point dried from CO_2_ and sputter-coated with gold. The morphology of the HCs was examined in a FEI Quanta 200 scanning electron microscope (ThermoFisher, Hillsboro, OR) and photographed.

#### Measurements of NLC and axial stiffness of OHCs

For recording NLC, isolated OHCs were bathed in extracellular solution containing (in mM) 120 NaCl, 20 TEA-Cl, 2 CoCl_2_, 2 MgCl_2_, 10 HEPES, and 5 glucose at pH 7.4. The internal solution contained (in mM): 140 CsCl, 2 MgCl_2_, 10 EGTA, and 10 HEPES at pH 7.4. The two-sine voltage stimulus protocol (10 mV peak at both 390.6 and 781.2 Hz) with subsequent fast Fourier transform-based admittance analysis (jClamp, version 15.1, SciSoft Company, Hew Heaven, CT) was used to measure membrane capacitance using jClamp software (Scisoft). Fits to the capacitance data were made in IgorPro (Wavemetrics, Lake Oswego, OR). The maximum charge transferred through the membrane’s electric field (Q_max_), the slope factor of the voltage dependence (a), the voltage at peak capacitance (V_pkcm_), and the linear membrane capacitance (C_lin_) were calculated.

The method for measuring axial stiffness is described previously (He et al., 2003; He and Dallos, 1999). In brief, the isolated OHC was drawn into a microchamber with ~75–80% of its length extruded. The tip of the glass fiber was brought against the cuticular plate of an OHC transverse to the cell’s long axis in such a way that the fiber’s lateral motion would compress or relax the cell. The cell’s axial stiffness (kc) was calculated by comparing the free movement of the tip of the fiber (Lf) of known stiffness (kf) with the loaded movement of the tip after it was rested against the surface of the cuticular plate (Lc), where: Kc = kf (Lf - L c)/Lc. Photodiode-based system was used to measure free fiber and loaded fiber motion (Jia and He, 2005).

### QUANTIFICATION AND STATISTICAL ANALYSIS

Means and standard deviations were calculated. Student’s t-test was used to determine statistical significance. Two-way ANOVA with multiple t-tests using the Holm-Sidak correction for multiple comparisons was also used to determine statistical significance. Probability (P) value ≤0.05 was regarded as significant. For transcriptome analysis, average expression (RPKM) values for each gene and SD were calculated. ANOVA False Discovery Rate-corrected p values < 0.10 was considered statistically significant. Differentially expressed genes were defined as those whose expression levels are above background with a Log2 fold change in expression ≥ |1| between the two ages (FDR p ≤ 0.10).

## Supplementary Material

1

2

3

4

## Figures and Tables

**Table:** KEY RESOURCES TABLE

REAGENT or RESOURCE	SOURCE	IDENTIFIER
Antibodies		
Rabbit polyclonal anti-DNM1	Novus Biologicals	Cat#: NBP2-48950
Rabbit polyclonal anti-KCNQ4	Sigma-Aldrich	Cat#: HPA018305; RRID:AB_1855556
Rabbit polyclonal anti-CBX3	Novus Biologicals	Cat#: NBP1-83228; RRID:AB_11037071
Rabbit polyclonal anti-SLC7A14	Sigma-Aldrich	Cat#: HPA045929; RRID:AB_2679501
Rabbit polyclonal anti-SLC26A5	Dr. Jing Zheng, Northwestern University	Dalllos et al., Neuron 58 (3): 333-339, 2008.
Mouse monoclonal anti-DNM3	Dr. Pietro De Camilli, Yale University	Raimondi et al., Neuron 70(6): 1100-1114, 2011.
Rabbit polyclonal anti-MYO7A	Proteus Biosciences	Cat# 25-6790; RRID: AB10015251
Goat anti-rabbit antibodies (secondary)	ThermoFisher	Cat#: A-11035; RRID:AB_2534093
Chemicals, peptides, and recombinant proteins		
RNAscope™ 2.5 HD Assay	Advanced Cell Diagnostics	Cat#: 322360
SMART-Seq v4 Ultra Low Input RNA Kit	TakaRa Bio USA Inc	Cat #: 634896, Lot#1712705A
Nextera Library preparation kit	Illumina	Cat#:15028211
RNA*later*	Thermo Fisher Scientific	Cat#: AM7020
Collagenase IV	Sigma-Aldrich	Cat#: C5138
Leibovitz’s L-15	Sigma-Aldrich	Cat#: L1518
Critical commercial assays		
RNAscope® Probe- Mm-Sod1	Advanced Cell Diagnostics	Cat#: 428581
RNAscope® Probe- Mm-Kcnq4	Advanced Cell Diagnostics	Cat#: 472271
RNAscope® Probe- Mm-Slc17a8	Advanced Cell Diagnostics	Cat#: 431261
RNAscope® Probe- Mm-C1ql1-C2	Advanced Cell Diagnostics	Cat#: 465081-C2
RNAscope® Probe- Mm-Tmc1	Advanced Cell Diagnostics	Cat#: 520911
RNAscope® Probe- Mm-Slc7a14	Advanced Cell Diagnostics	Cat#: 544781
RNAscope® Probe- Mm-Otof	Advanced Cell Diagnostics	Cat#: 485671
RNAscope® Probe- Mm-Chrna9	Advanced Cell Diagnostics	Cat#: 525921
RNAscope® Probe- Mm-Chrna10	Advanced Cell Diagnostics	Cat#: 525911
RNAscope® Probe- Mm-Clu	Advanced Cell Diagnostics	Cat#: 427891
RNAscope® Probe- Mm-Foxo3	Advanced Cell Diagnostics	Cat#: 485321
RNAscope® Probe- Mm-Jund-C1	Advanced Cell Diagnostics	Cat#: 1031951
Deposited data		
Hair cell transcriptomes (two time points: 9 and 26 months old)	This paper	https://www.ncbi.nlm.nih.gov/geo/query/acc.cgi?acc=GSE153882
Transcriptomes of cochlear stria vascularis (two time points: 9 months and 26 months)	This paper	https://www.ncbi.nlm.nih.gov/geo/query/acc.cgi?acc=GSE154833
Experimental models: Organisms/strains		
Mouse, CBA/J	The Jackson Laboratory	www.jax.org
Software and algorithms		
CLC Genomics Workbench software	Qiagen CLC Genomics	https://digitalinsights.qiagen.com
iDEP 0.93	http://bioinformatics.sdstate.edu/idep/	http://bioinformatics.sdstate.edu/idep/
ClueGO v. 2.5.7	Cytoscape	https://apps.cytoscape.org/apps/cluego
FastQC	https://basespace.illumina.com/home/index	https://basespace.illumina.com/home/index
ImageJ	NIH	https://imagej.nih.gov/ij/
Adobe Photoshop and Illustrator	Adobe	https://www.adobe.com/
Imaris	Oxford Instruments	https://imaris.oxinst.com/
pClamp v. 10.2	Molecular Devices	https://www.moleculardevices.com/products/axon-patch-clamp-system
jClamp 11.1	SciSoft Company	http://www.scisoftco.com/
Other		
Ensembl database	http://uswest.ensembl.org/Mus_musculus/Info/Index	http://uswest.ensembl.org/Mus_musculus/Info/Index
AmiGO	http://amigo.geneontology.org/amigo	http://amigo.geneontology.org/amigo
gEAR	www.umgear.org	www.umgear.org
HGNC database	https://www.genenames.org/	https://www.genenames.org/

## Highlights

- Transcriptomic analysis of aging hair cells shows common molecular aging hallmarks
- Age-related transcriptional changes also include hair-cell-specific genes
- Cytological aging is manifested as degeneration of stereocilia and cell soma
- Hair cell degeneration preceding cell loss contributes to age-related hearing loss

## References

[R1] Ao X, Zou L, and Wu Y (2014). Regulation of autophagy by the Rab GTPase network. Cell Death Differ. 21, 348–358. 10.1038/cdd.2013.187. 24440914 PMC3921601

[R2] Balistreri CR, Madonna R, Melino G, and Caruso C (2016). The emerging role of Notch pathway in ageing: focus on the related mechanisms in age-related diseases. Ageing Res. Rev. 29, 50–65. 10.1016/j.arr.2016.06.004. 27328278

[R3] Barr-Gillespie P-G (2015). Assembly of hair bundles, an amazing problem for cell biology. Mol. Biol. Cell 26, 2727–2732. 10.1091/mbc.E14-04-0940. 26229154 PMC4571333

[R4] Belyantseva IA, Boger ET, Naz S, Frolenkov GI, Sellers JR, Ahmed ZM, Griffith AJ, and Friedman TB (2005). Myosin-XVa is required for tip localization of whirlin and differential elongation of hair-cell stereocilia. Nat. Cell Biol. 7, 148–156. 10.1038/ncb1219. 15654330

[R5] Bindea G, Mlecnik B, Hackl H, Charoentong P, Tosolini M, Kirilovsky A, Fridman W-H, Pagès F, Trajanoski Z, and Galon J (2009). ClueGO: a Cytoscape plug-in to decipher functionally grouped gene ontology and pathway annotation networks. Bioinformatics 25, 1091–1093. 10.1093/bioinformatics/btp101. 19237447 PMC2666812

[R6] Brownell WE, Bader CR, Bertrand D, and de Ribaupierre Y (1985). Evoked mechanical responses of isolated cochlear outer hair cells. Science 227, 194–196. 10.1126/science.3966153. 3966153

[R7] Bullen A, Forge A, Wright A, Richardson GP, Goodyear RJ, and Taylor R (2020). Ultrastructural defects in stereocilia and tectorial membrane in aging mouse and human cochleae. J. Neurosci. Res. 98, 1745–1763. 10.1002/jnr.24556. 31762086

[R8] Dallos P, Wu X, Cheatham MA, Gao J, Zheng J, Anderson CT, Jia S, Wang X, Cheng WHY, Sengupta S, (2008). Prestin-based outer hair cell motility is necessary for mammalian cochlear amplification. Neuron 58, 333–339. 10.1016/j.neuron.2008.02.028. 18466744 PMC2435065

[R9] Davie K, Janssens J, Koldere D, De Waegeneer M, Pech U, Kreft Ł, Aibar S, Makhzami S, Christiaens V, Bravo González-Blas C, (2018). A single-cell transcriptome atlas of the aging Drosophila brain. Cell 174, 982–998.e20. 10.1016/j.cell.2018.05.057. 29909982 PMC6086935

[R10] Du H, Ye C, Wu D, Zang Y-Y, Zhang L, Chen C, He X-Y, Yang J-J, Hu P, Xu Z, (2020). The cation channel TMEM63B is an osmosensor required for hearing. Cell Rep. 31, 107596. 10.1016/j.celrep.2020.107596. 32375046

[R11] Ewing B, Hillier L, Wendl MC, and Green P (1998). Base-calling of automated sequencer traces Using Phred. I. Accuracy assessment. Genome Res. 8, 175–185. 10.1101/gr.8.3.175. 9521921

[R12] Frisina RD (2009). Age-related hearing loss. Ann. N. Y. Acad. Sci. 1170, 708–717. 10.1111/j.1749-6632.2009.03931.x. 19686217

[R13] Gates GA, Cooper JC, Kannel WB, and Miller NJ (1990). Hearing in the elderly: the Framingham cohort, 1983-1985. Part I. Basic audiometric test results. Ear Hear. 11, 247–256. 2210098

[R14] Giffen KP, Li Y, Liu HZ, Zhao XC, Zhang CJ, Shen RJ, Wang TY, Janesick A, Chen BP, Gong SS, Kachar B, (2022). Mutation of SLC7A14 causes auditory neuropathy and retinitis pigmentosa mediated by lysosomal dysfunction. Sci. Adv. 8, eabk0942. 10.1126/sciadv.abk0942. 35394837 PMC8993119

[R15] Gilels F, Paquette ST, Zhang J, Rahman I, and White PM (2013). Mutation of Foxo3 causes adult onset auditory neuropathy and alters cochlear synapse architecture in mice. J. Neurosci. 33, 18409–18424. 10.1523/JNEUROSCI.2529-13.2013. 24259566 PMC6618809

[R16] Hall H, Medina P, Cooper DA, Escobedo SE, Rounds J, Brennan KJ, Vincent C, Miura P, Doerge R, and Weake VM (2017). Transcriptome profiling of aging Drosophila photoreceptors reveals gene expression trends that correlate with visual senescence. BMC Genomics 18, 894. 10.1186/s12864-017-4304-3. 29162050 PMC5698953

[R17] Hammond TR, Dufort C, Dissing-Olesen L, Giera S, Young A, Wysoker A, Walker AJ, Gergits F, Segel M, Nemesh J, (2019). Single-cell RNA sequencing of microglia throughout the mouse lifespan and in the injured brain reveals complex cell-state changes. Immunity 50, 253–271.e6. 10.1016/j.immuni.2018.11.004. 30471926 PMC6655561

[R18] He DZZ, and Dallos P (1999). Somatic stiffness of cochlear outer hair cells is voltage-dependent. Proc. Natl. Acad. Sci. U S A. 96,8223–8228. 10.1073/pnas.96.14.8223. 10393976 PMC22216

[R19] He DZZ, Jia S, and Dallos P (2003). Prestin and the dynamic stiffness of cochlear outer hair cells. J. Neurosci. 23, 9089–9096. 10.1523/JNEUROSCI.23-27-09089.2003. 14534242 PMC6740818

[R20] He DZZ, Jia S, Sato T, Zuo J, Andrade LR, Riordan GP, and Kachar B (2010). Changes in plasma membrane structure and electromotile properties in prestin deficient outer hair cells. Cytoskeleton 67, 43–55. 10.1002/cm.20423. 20169529 PMC2842980

[R21] Health, United States, 1994. U.S. Dept. of Health, Education, and Welfare, Public Health Service, Health Resources Administration, National Center for Health Statistics; Washington, D.C. https://lccn.loc.gov/76641496

[R22] van Heemst D (2010). Insulin, IGF-1 and longevity. Aging Dis. 1, 147–157. 22396862 PMC3295030

[R23] Jia S, and He DZZ (2005). Motility-associated hair-bundle motion in mammalian outer hair cells. Nat. Neurosci. 8, 1028–1034. 10.1038/nn1509. 16041370

[R24] Jongkamonwiwat N, Ramirez MA, Edassery S, Wong ACY, Yu J, Abbott T, Pak K, Ryan AF, and Savas JN (2020). Noise exposures causing hearing loss generate proteotoxic stress and activate the proteostasis network. Cell Rep. 33, 108431. 10.1016/j.celrep.2020.108431. 33238128 PMC7722268

[R25] Kawashima Y, Géléoc GSG, Kurima K, Labay V, Lelli A, Asai Y, Makishima T, Wu DK, Della Santina CC, Holt JR, and Griffith AJ (2011). Mechanotransduction in mouse inner ear hair cells requires transmembrane channel–like genes. J. Clin. Invest. 121, 4796–4809. 10.1172/JCI60405. 22105175 PMC3223072

[R26] Kimura KD (1997). daf-2, an insulin receptor-like gene that regulates longevity and diapause in Caenorhabditis elegans. Science 277, 942–946. 10.1126/science.277.5328.942. 9252323

[R27] Krey JF, Sherman NE, Jeffery ED, Choi D, and Barr-Gillespie PG (2015). The proteome of mouse vestibular hair bundles over development. Sci. Data 2, 150047. 10.1038/sdata.2015.47. 26401315 PMC4570149

[R28] Kubisch C, Schroeder BC, Friedrich T, Lütjohann B, El-Amraoui A, Marlin S, Petit C, and Jentsch TJ (1999). KCNQ4, a novel potassium channel expressed in sensory outer hair cells, is mutated in dominant deafness. Cell 96, 437–446. 10.1016/S0092-8674(00)80556-5. 10025409

[R29] Kwon SK, and Workman J.l. (2008). The heterochromatin protein 1 (HP1) family: put away a bias toward HP1. Mol. Cells 26, 217–227. 18664736

[R30] Lauer AM, Fuchs PA, Ryugo DK, and Francis HW (2012). Efferent synapses return to inner hair cells in the aging cochlea. Neurobiol. Aging 33,2892–2902. 10.1016/j.neurobiolaging.2012.02.007. 22405044 PMC3398161

[R31] Lee S-H, Lee J-H, Lee H-Y, and Min K-J (2019). Sirtuin signaling in cellular senescence and aging. BMB Rep. 52, 24–34. 10.5483/BMBRep.2019.52.1.290. 30526767 PMC6386230

[R32] Li Y, Liu H, Barta CL, Judge PD, Zhao L, Zhang WJ, Gong S, Beisel KW, and He DZZ (2016). Transcription factors expressed in mouse cochlear inner and outer hair cells. PLoS One 11, e0151291. 10.1371/journal.pone.0151291. 26974322 PMC4790917

[R33] Li Y, Jia S, Liu H, Tateya T, Guo W, Yang S, Beisel KW, and He DZZ (2018a). Characterization of hair cell-like cells converted from supporting cells after Notch inhibition in cultures of the organ of Corti from neonatal gerbils. Front. Cell Neurosci. 12, 73. 10.3389/fncel.2018.00073. 29662441 PMC5890164

[R34] Li Y, Liu H, Giffen KP, Chen L, Beisel KW, and He DZZ (2018b). Transcriptomes of cochlear inner and outer hair cells from adult mice. Sci. Data 5, 180199. 10.1038/sdata.2018.199. 30277483 PMC6167952

[R35] Liang X, Qiu X, Dionne G, Cunningham CL, Pucak ML, Peng G, Kim Y-H, Lauer A, Shapiro L, and Müller U (2021). CIB2 and CIB3 are auxiliary subunits of the mechanotransduction channel of hair cells. Neuron 109, 2131–2149.e15. 10.1016/j.neuron.2021.05.007. 34089643 PMC8374959

[R36] Liberman MC, Gao J, He DZZ, Wu X, Jia S, and Zuo J (2002). Prestin is required for electromotility of the outer hair cell and for the cochlear amplifier. Nature 419, 300–304. 12239568 10.1038/nature01059

[R37] Lin FR, and Albert M (2014). Hearing loss and dementia–who is listening? Aging Ment. Health 18, 671–673. 10.1080/13607863.2014.915924. 24875093 PMC4075051

[R38] Lin K, Hsin H, Libina N, and Kenyon C (2001). Regulation of the Caenorhabditis elegans longevity protein DAF-16 by insulin/IGF-1 and germline signaling. Nat. Genet. 28, 139–145. 10.1038/88850. 11381260

[R39] Liu H, Pecka JL, Zhang Q, Soukup GA, Beisel KW, and He DZZ (2014). Characterization of transcriptomes of cochlear inner and outer hair cells. J. Neurosci. 34, 11085–11095. 10.1523/JNEUROSCI.1690-14.2014. 25122905 PMC4131018

[R40] Liu H, Li Y, Chen L, Zhang Q, Pan N, Nichols DH, Zhang WJ, Fritzsch B, and He DZZ (2016). Organ of Corti and stria vascularis: is there an interdependence for survival? PLoS One 11,e0168953. 10.1371/journal.pone.0168953. 28030585 PMC5193441

[R41] Liu H, Giffen KP, Grati M, Morrill SW, Li Y, Liu X, Briegel KJ, and He DZ (2021). Transcription co-factor LBH is necessary for the survival of cochlear hair cells. J. Cell Sci. 134. 10.1242/jcs.254458. PMC8077238 33674448

[R42] López-Otín C, Blasco MA, Partridge L, Serrano M, and Kroemer G (2013). The hallmarks of aging. Cell 153, 1194–1217. 10.1016/j.cell.2013.05.039. 23746838 PMC3836174

[R43] Lu T, Pan Y, Kao S-Y, Li C, Kohane I, Chan J, and Yankner BA (2004). Gene regulation and DNA damage in the ageing human brain. Nature 429, 883–891. 15190254 10.1038/nature02661

[R44] Manor U, Disanza A, Grati M, Andrade L, Lin H, Di Fiore PP, Scita G, and Kachar B (2011). Regulation of stereocilia length by myosin XVa and whirlin depends on the actin-regulatory protein Eps8. Curr. Biol. 21, 167–172. 10.1016/j.cub.2010.12.046. 21236676 PMC3040242

[R45] Mao Z, Zhao L, Pu L, Wang M, Zhang Q, and He DZZ (2013). How well can centenarians hear? PLoS One 8, e65565. 10.1371/journal.pone.0065565. 23755251 PMC3673943

[R46] Milon B, Shulman ED, So KS, Cederroth CR, Lipford EL, Sperber M, Sellon JB, Sarlus H, Pregernig G, Shuster B, (2021). A cell-type-specific atlas of the inner ear transcriptional response to acoustic trauma. Cell Rep. 36, 109758. 10.1016/j.celrep.2021.109758. 34592158 PMC8709734

[R47] Moore LD, Le T, and Fan G (2013). DNA methylation and its basic function. Neuropsychopharmacology 38, 23–38. 10.1038/npp.2012.112. 22781841 PMC3521964

[R48] Morris BJ, Willcox DC, Donlon TA, and Willcox BJ (2015). *FOXO3*: a major gene for human longevity - a mini-review. Gerontology 61, 515–525. 10.1159/000375235. 25832544 PMC5403515

[R49] Narayanan P, Chatterton P, Ikeda A, Ikeda S, Corey DP, Ervasti JM, and Perrin BJ (2015). Length regulation of mechanosensitive stereocilia depends on very slow actin dynamics and filament-severing proteins. Nat. Commun. 6, 6855. 10.1038/ncomms7855. 25897778 PMC4523390

[R50] Ohlemiller KK (2004). Age-related hearing loss: the status of Schuknecht’s typology. Curr. Opin. Otolaryngol. Head Neck Surg. 12, 439–443. 10.1097/01.moo.0000134450.99615.22. 15377958

[R51] Ohlemiller KK (2006). Contributions of mouse models to understanding of age- and noise-related hearing loss. Brain Res. 1091, 89–102. 10.1016/j.brainres.2006.03.017. 16631134

[R52] Ohlemiller KK, Dahl AR, and Gagnon PM (2010). Divergent aging characteristics in CBA/J and CBA/CaJ mouse cochleae. J. Assoc. Res. Otolaryngol. 11, 605–623. 10.1007/s10162-010-0228-1. 20706857 PMC2975886

[R53] Qi Y, Xiong W, Yu S, Du Z, Qu T, He L, Wei W, Zhang L, Liu K, Li Y, (2021). Deletion of C1ql1 causes hearing loss and abnormal auditory nerve fibers in the mouse cochlea. Front. Cell Neurosci. 15, 713651. 34512267 10.3389/fncel.2021.713651 PMC8424102

[R54] Sancak Y, Peterson TR, Shaul YD, Lindquist RA, Thoreen CC, Bar-Peled L, and Sabatini DM (2008). The rag GTPases bind raptor and mediate amino acid signaling to mTORC1. Science 320,1496–1501. 10.1126/science.1157535. 18497260 PMC2475333

[R55] Santos-Sacchi J (1991). Reversible inhibition of voltage-dependent outer hair cell motility and capacitance. J. Neurosci. 11, 3096–3110. 10.1523/JNEUROSCI.11-10-03096.1991. 1941076 PMC6575435

[R56] Schulte BA, and Schmiedt RA (1992). Lateral wall Na, K-ATPase and endocochlear potentials decline with age in quiet-reared gerbils. Hear. Res. 61, 35–46. 10.1016/0378-5955(92)90034-K. 1326507

[R57] Sergeyenko Y, Lall K, Liberman MC, and Kujawa SG (2013). Age-related cochlear synaptopathy: an early-onset contributor to auditory functional decline. J. Neurosci. 33, 13686–13694. 10.1523/JNEUROSCI.1783-13.2013. 23966690 PMC3755715

[R58] Sha S-H, Kanicki A, Dootz G, Talaska AE, Halsey K, Dolan D, Altschuler R, and Schacht J (2008). Age-related auditory pathology in the CBA/J mouse. Hear. Res. 243, 87–94. 10.1016/j.heares.2008.06.001. 18573325 PMC2577824

[R59] Smallwood A, Hon GC, Jin F, Henry RE, Espinosa JM, and Ren B (2012). CBX3 regulates efficient RNA processing genome-wide. Genome Res. 22, 1426–1436. 10.1101/gr.124818.111. 22684280 PMC3409256

[R60] Spongr VP, Flood DG, Frisina RD, and Salvi RJ (1997). Quantitative measures of hair cell loss in CBA and C57BL/6 mice throughout their life spans. J. Acoust. Soc. Am. 101, 3546–3553. 10.1121/1.418315. 9193043

[R61] Stepanyan R, and Frolenkov GI (2009). Fast adaptation and Ca2+ sensitivity of the mechanotransducer require myosin-XVa in inner but not outer cochlear hair cells. J. Neurosci. 29, 4023–4034. 10.1523/JNEUROSCI.4566-08.2009. 19339598 PMC2702482

[R62] Tacutu R, Thornton D, Johnson E, Budovsky A, Barardo D, Craig T, Diana E, Lehmann G, Toren D, Wang J, (2018). Human ageing genomic resources: new and updated databases. Nucleic Acids Res. 46, D1083–D1090. 10.1093/nar/gkx1042. 29121237 PMC5753192

[R63] The Tabula Muris Consortium; Almanzar N, Antony J, Baghel AS, Bakerman I, Bansal I, Barres BA, Beachy PA, Berdnik D, Bilen B, Brownfield D, and Cain C (2020). A single-cell transcriptomic atlas characterizes ageing tissues in the mouse. Nature 583, 590–595. 10.1038/s41586-020-2496-1. 32669714 PMC8240505

[R64] Vijg J, and Suh Y (2013). Genome instability and aging. Annu. Rev. Physiol. 75, 645–668. 10.1146/annurev-physiol-030212-183715. 23398157

[R65] Weichhart T (2018). mTOR as regulator of lifespan, aging, and cellular senescence: a mini-review. Gerontology 64, 127–134. 10.1159/000484629. 29190625 PMC6089343

[R66] Weitzman JB, Fiette L, Matsuo K, and Yaniv M (2000). JunD protects cells from p53-dependent senescence and apoptosis. Mol. Cell 6, 1109–1119. 10.1016/S1097-2765(00)00109-X. 11106750

[R67] Wu P, O’Malley JT, de Gruttola V, and Liberman MC (2020). Age-related hearing loss is dominated by damage to inner ear sensory cells, not the cellular battery that powers them. J. Neurosci. 40, 6357–6366. 10.1523/JNEUROSCI.0937-20.2020. 32690619 PMC7424870

[R68] Ximerakis M, Lipnick SL, Innes BT, Simmons SK, Adiconis X, Dionne D, Mayweather BA, Nguyen L, Niziolek Z, Ozek C, (2019). Single-cell transcriptomic profiling of the aging mouse brain. Nat. Neurosci. 22,1696–1708. 10.1038/s41593-019-0491-3. 31551601

[R69] Xiong W, Grillet N, Elledge HM, Wagner TFJ, Zhao B, Johnson KR, Kazmierczak P, and Müller U (2012). TMHS is an integral component of the mechanotransduction machinery of cochlear hair cells. Cell 151, 1283–1295. 10.1016/j.cell.2012.10.041. 23217710 PMC3522178

[R70] Yi W, Lu Y, Zhong S, Zhang M, Sun L, Dong H, Wang M, Wei M, Xie H, Qu H, (2020). A single-cell transcriptome atlas of the aging human and macaque retina. Natl. Sci. Rev. nwaa179. 10.1093/nsr/nwaa179. 34691611 PMC8288367

[R71] Zhang MJ, Pisco AO, Darmanis S, and Zou J (2021). Mouse aging cell atlas analysis reveals global and cell type-specific aging signatures. eLife 10, e62293. 10.7554/eLife.62293. 33847263 PMC8046488

[R72] Zhao B, Wu Z, Grillet N, Yan L, Xiong W, Harkins-Perry S, and Müller U (2014). TMIE is an essential component of the mechanotransduction machinery of cochlear hair cells. Neuron 84, 954–967. 10.1016/j.neuron.2014.10.041. 25467981 PMC4258123

[R73] Zheng J, Shen W, He DZZ, Long KB, Madison LD, and Dallos P (2000). Prestin is the motor protein of cochlear outer hair cells. Nature 405, 149–155. 10.1038/35012009. 10821263

