# Supplementary material for: Molecular and cytological profiling of biological aging of mouse cochlear inner and outer hair cells
Source: Cell Rep. Author manuscript; Available in PMC 2022 May 5. (PMC9069708; doi:10.1016/j.celrep.2022.110665)
Supplement: 1 [file NIHMS1798659-supplement-1.pdf]

Cell Reports, Volume *39*

Supplemental information

Molecular and cytological profiling of biological aging of mouse cochlear inner and outer hair cells

Huizhan Liu, Kimberlee P. Giffen, Lei Chen, Heidi J. Henderson, Talia A. Cao, Grant A. Kozeny, Kirk W. Beisel, Yi Li, and David Z. He

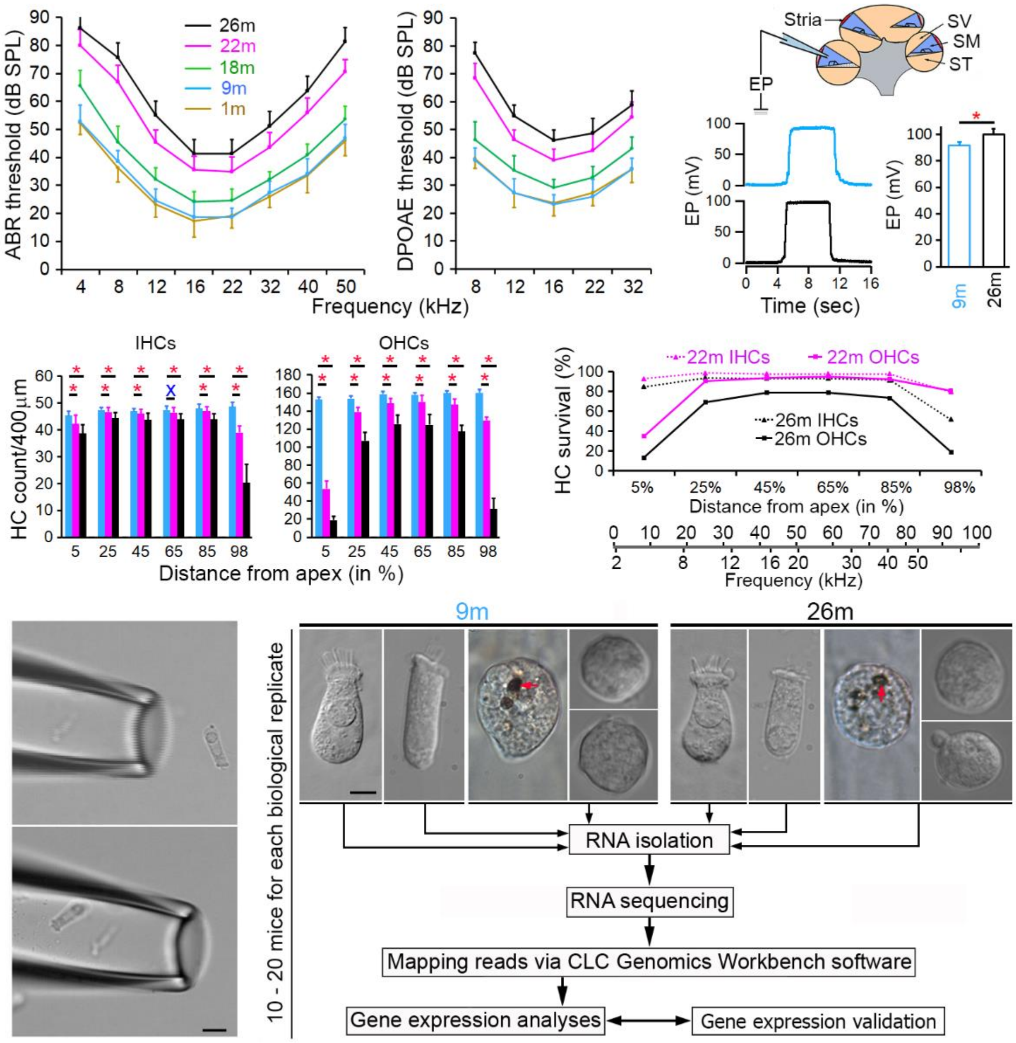

Figure S1: Auditory function and HC status during aging and overview of the study design for cell type-specific RNA-seq. Related to Figure 1.

(A,B) Mean ABR and DPOAE thresholds of 1-, 9- 18-, 22- and 26-months-old CBA/J mice (n =11 mice for 1-, 9- and 18m-old; n = 10 for 22m and 8 for 26m). A two-way ANOVA with multiple t-tests showed no statistical significance in ABR and DPOAE thresholds between 1 and 9 months (p values all ≥ 0.05). Significant differences ($p < 0.05$) were found at all frequencies at 18m and onward with reference to the thresholds at 9m. (C) EP measured from mice aged 9 (n = 7) and 26 months (n = 6). Significant difference (p = 0.0025) was found (marked by a red asterisk). (D) HC count at 6 different cochlear locations at 9, 22 and 26 months. Red asterisk marks statistical significance ($p<0.05$) while blue X marks no significance. (E) Surviving HCs at the different cochlear locations and their corresponding frequencies. (F) Suction pipette technique used to collect isolated HCs and stria cells. Bar: 10 μm. (G) An overview of the study design for cell collection and RNA-sequencing together with representative images of isolated IHCs, OHCs and cells from stria from 9m- and 26m-old cochleae. The stria vascularis contains three different cell types. Only melanocytes are identifiable with melanin (marked with red arrow) inside the cells. Bar: 5 μm.

A

Enrichment FDR	nGenes	Downregulated Pathways	UP OHCs	Down OHCs	UP IHCs	Down IHCs
		ShinyGO enriched BP of DEGs in IP OHCs post-noise 24hr v 0hr pulled from gEAR	ShinyGO enriched BP of DEGs in Aging OHCs and IHCs (Figures 1 and 2)			
7.66E-10	47	Anterograde trans-synaptic signaling		x		
8.14E-12	58	Cation transmembrane transport				
1.18E-11	69	Cation transport		x		
4.99E-10	104	Cell development				
7.66E-10	47	Chemical synaptic transmission				
6.84E-14	90	Generation of neurons				
1.80E-13	22	Glutamate receptor signaling pathway				
7.66E-10	52	Inorganic ion transmembrane transport		x		
1.34E-13	71	Ion transmembrane transport				
1.23E-11	85	Ion transport		x		
1.71E-09	53	Metal ion transport				
1.28E-18	126	Nervous system development		x		
3.08E-13	92	Neurogenesis	x	x		
1.34E-13	74	Neuron development				
9.35E-13	81	Neuron differentiation		x		
9.99E-10	60	Neuron projection development				
9.99E-10	45	Neuron projection morphogenesis				
1.71E-09	45	Plasma membrane bounded cell projection morphogenesis				
7.66E-10	76	Plasma membrane bounded cell projection organization				
1.34E-13	45	Regulation of ion transmembrane transport		x		
1.10E-14	58	Regulation of ion transport				
1.10E-14	45	Regulation of membrane potential				
1.64E-09	59	Regulation of nervous system development				
4.28E-13	48	Regulation of transmembrane transport		x		
7.66E-10	86	Regulation of transport				
3.71E-11	40	Synapse organization				
1.59E-10	50	Synaptic signaling	x			
9.99E-10	167	System development				
9.99E-10	47	Trans-synaptic signaling				
2.57E-10	77	Transmembrane transport		x		

Enrichment FDR	nGenes	Upregulated Pathways	UP OHCs	Down OHCs	UP IHCs	Down IHCs
2.64E-16	135	Cellular response to chemical stimulus				
2.70E-19	66	Cellular response to cytokine stimulus	x			
2.41E-13	16	Cellular response to interferon-beta				
3.19E-16	116	Cellular response to organic substance				
2.34E-12	38	Cytokine-mediated signaling pathway				
2.21E-29	112	Defense response	x			
8.20E-25	83	Defense response to other organism				
2.62E-14	55	Immune effector process				
1.11E-21	94	Immune response				
5.92E-24	133	Immune system process	x			
5.85E-12	50	Inflammatory response	x			
2.16E-23	69	Innate immune response		x		x
3.33E-30	116	Interspecies interaction between organisms				
2.34E-12	38	Positive regulation of response to external stimulus				
4.09E-10	47	Regulation of cytokine production				
2.01E-11	44	Regulation of defense response				
1.49E-10	68	Regulation of immune system process	x			
1.06E-12	59	Regulation of response to external stimulus				
2.98E-21	66	Response to bacterium				
4.99E-28	105	Response to biotic stimulus				
1.85E-21	74	Response to cytokine			x	
3.40E-28	104	Response to external biotic stimulus				
5.30E-23	140	Response to external stimulus				
1.16E-15	19	Response to interferon-beta				
4.20E-13	24	Response to interferon-gamma				
8.33E-17	145	Response to organic substance				
3.40E-28	104	Response to other organism				
8.51E-21	162	Response to stress		x		
8.42E-14	36	Response to virus				
1.06E-12	38	Symbiotic process				

B

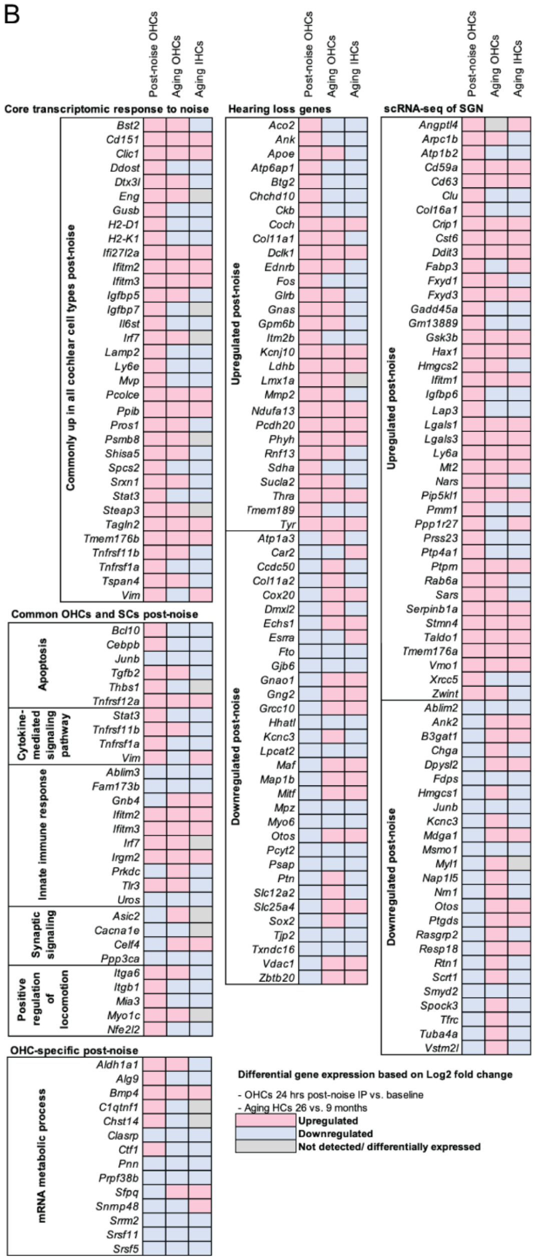

Figure S2: Differential gene expression in post-noise and aging HCs. Related to Figure 4**.**
(A) The differentially expressed genes from post-noise OHCs were downloaded from the gEAR database. Fold change values were based on gene expression 24 hrs post-noise exposure compared to baseline IP Prestin ribo-tagged OHCs. Gene lists were input into ShinyGO and the top enriched biological processes (BP) were identified. This lists of the top 30 up and downregulated processes were compared to the IHC and OHC lists of enriched GO biological processes in figures 1 and 2, respectively. Common processes among cell types are identified. (B) The trend of expression in a subset of genes after noise exposure compared to the differential expression trend in aging OHCs and IHCs. Several genes show a common trend in expression and overall the OHCs have more in common across the two studies. The groups of genes shown were identified in the Milon et al. study (2021) including: core transcriptional response to noise, common cochlear HC and support cell response to noise, unique OHC response to noise, and hearing loss phenotype-associated genes. Differential gene expression in post- noise spiral ganglion neurons (single-cell RNAseq) was also compared to aging HCs which revealed similar trends in downregulated genes between spiral ganglion neurons and IHCs.
